# Does the COVID-19 pandemic impact parents’ and adolescents’ well-being? An EMA-study on daily affect and parenting

**DOI:** 10.1371/journal.pone.0240962

**Published:** 2020-10-16

**Authors:** Loes H. C. Janssen, Marie-Louise J. Kullberg, Bart Verkuil, Noa van Zwieten, Mirjam C. M. Wever, Lisanne A. E. M. van Houtum, Wilma G. M. Wentholt, Bernet M. Elzinga

**Affiliations:** 1 Department of Clinical Psychology, Institute of Psychology, Leiden University, Leiden, The Netherlands; 2 Leiden Institute for Brain and Cognition (LIBC), Leiden University, Leiden, The Netherlands; Sapienza - University of Roma, Italy, ITALY

## Abstract

Due to the COVID- 19 outbreak in the Netherlands (March 2020) and the associated social distancing measures, families were enforced to stay at home as much as possible. Adolescents and their families may be particularly affected by this enforced proximity, as adolescents strive to become more independent. Yet, whether these measures impact emotional well-being in families with adolescents has not been examined. In this ecological momentary assessment study, we investigated if the COVID-19 pandemic affected positive and negative affect of parents and adolescents and parenting behaviors (warmth and criticism). Additionally, we examined possible explanations for the hypothesized changes in affect and parenting. To do so, we compared daily reports on affect and parenting that were gathered during two periods of 14 consecutive days, once before the COVID-19 pandemic (2018–2019) and once during the COVID-19 pandemic. Multilevel analyses showed that only parents’ negative affect increased as compared to the period before the pandemic, whereas this was not the case for adolescents’ negative affect, positive affect and parenting behaviors (from both the adolescent and parent perspective). In general, intolerance of uncertainty was linked to adolescents’ and parents’ negative affect and adolescents’ positive affect. However, Intolerance of uncertainty, nor any pandemic related characteristics (i.e. living surface, income, relatives with COVID-19, hours of working at home, helping children with school and contact with COVID-19 patients at work) were linked to the increase of parents’ negative affect during COVID-19. It can be concluded that on average, our sample (consisting of relatively healthy parents and adolescents) seems to deal fairly well with the circumstances. The substantial heterogeneity in the data however, also suggest that whether or not parents and adolescents experience (emotional) problems can vary from household to household. Implications for researchers, mental health care professionals and policy makers are discussed.

## Introduction

**Fig 1:**
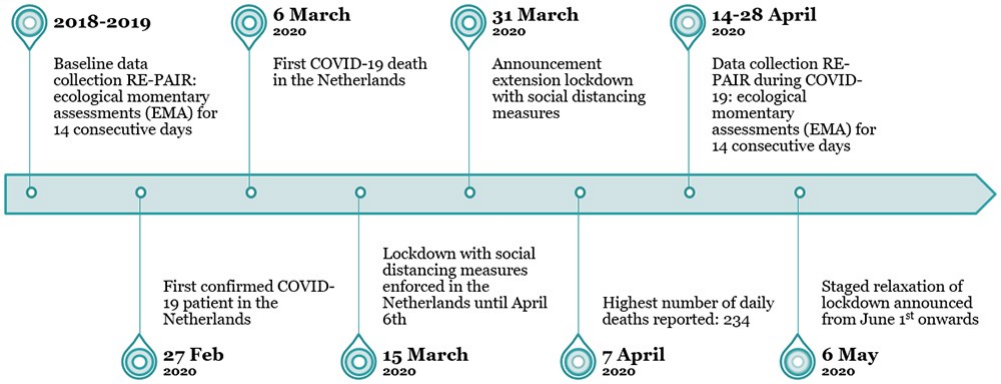
Timeline of study period.

Since March 2020, the coronavirus disease 2019 (COVID-19) is referred to as a pandemic by the World Health Organization [1]. To slow the spread of COVID-19, national governments have taken radical measures to minimize social interactions by closing public places, demanding people to keep physical distance and stay at home and—in some countries—by enforcing ‘full lockdown’. In the Netherlands, at March 15^th^ 2020, measures of social distancing enforced all Dutch citizens to stay home and work remotely as much as possible, public spaces (e.g. schools, offices, parts of public transport, theatres) were closed and public gatherings were prohibited (see Fig 1 for a timeline). These measures of social distancing (a so-called ‘lockdown') created drastic changes in daily social life; distinct domains such as family life, school, and work suddenly coincided and families faced an unforeseen increase in hours spent together under the same roof. Adolescents and their families may be particularly affected by this enforced proximity, as adolescents strive to become independent and focus more on socializing and spending time with friends rather than with their families [2, 3]. To that end, this study aimed to investigate well-being of adolescents and their parents and parenting behaviors during the COVID-19 pandemic and explored daily difficulties and helpful activities during the COVID-19 pandemic linked to their well-being.

For some families, spending more time together during a lockdown may bring family members closer towards each other and foster a sense of well-being. However, several factors that are emblematic for the COVID-19 crisis, such as financial insecurity, concerns about own and others’ health, uncertainty about quarantine duration, lack of social and physical activities, and boredom have all frequently been shown to negatively affect a person’s mood and mental well-being [4–8]. Moreover, parents and adolescents may also experience stress because they are faced with more daily hassles (e.g. a suboptimal work or school environment) and additional tasks (e.g. parents homeschooling their children or caring for significant others). Previous studies have shown that the impact of these quarantine related factors on mental health outcomes (e.g. depressive symptoms, anxiety, and PTSD) can be wide-ranging, substantial and long-lasting (see review of Brooks et al. [9]). As a consequence, these confinements may also lead to more tension, irritability, family conflicts, and at worse, domestic violence or child abuse [10].

One of the key questions that have been raised by governmental agencies and health care workers is to what extent the COVID-19 pandemic and the associated distancing measures affect families’ well-being and parenting behaviors. In this study, Dutch adolescents and their parents filled in 14 days of ecological momentary assessments (EMA; [11]) twice, *before* the COVID-19 outbreak (2018–2019) and also *during* the COVID-19 pandemic (14–28 April 2020). In addition, we asked parents and adolescents about daily difficulties and helpful activities during the COVID-19 pandemic that possibly influenced their affect in positive and negative ways. This enabled us to investigate how and to what extent well-being and parenting behaviors in daily life were impacted by the COVID-19 pandemic and the related social distancing measures. Gaining more insight into these processes, our findings can contribute to formulating recommendations for policy makers and mental health professionals.

### Positive and negative affect in daily life

Individuals’ affect states are not one-dimensional and static in nature, but can fluctuate from moment to moment in response to other individuals and external circumstances (e.g., [12]). Positive and negative affect reflect a persons’ momentary mood state. Both positive and negative affect have implications for health and well-being over time for adults and adolescents [13–18]. Positive affect predominantly generates action, motivation, social connectedness and cognitive flexibility, whereas negative affect might result in actions such as avoidance, attack, or expel [19, 20]. Using momentary assessments enabled us to identify the potential impact of the pandemic on parents’ and adolescents’ positive and negative affect in daily life without the potential bias of retrospective recall.

### Parenting

The COVID-19 pandemic and the related social measures might also impact parenting behaviors, such as the amount of expressed warmth and criticism. Parental warmth is typically considered as one of the primary dimensions of sensitive parenting behavior and can include acceptance, support, and positive involvement towards the child [21]. Parental criticism can be defined as expressing negativity, disapproval, or dissatisfaction to a child [22]. Psychological distress related to the COVID-19 pandemic may influence parenting behaviors, with parents being more emotionally withdrawn or critical and irritated, instead of being supportive, sensitive and encouraging to the child [23].

Previous studies have shown that especially positive mood of family members is closely related to warm family interactions, whereas negative mood is related to withdrawal from interactions [19, 24–26]. However, no prior studies have examined the effects of a situation comparable to the current COVID-19 pandemic on parenting. Therefore, in addition to its impact on affect, we also aimed to investigate the impact of the COVID-19 pandemic and its consequences on parental warmth and criticism in daily life. Since parenting is a dynamic process [16], we will examine day-to-day parental warmth and criticism. Furthermore, as perspectives from parents and adolescents on parenting might differ (e.g., [27]), we examined both the parent and adolescent perspective on parental warmth and criticism.

### Intolerance of uncertainty

A crucial aspect of unforeseen stressful situations, such as the COVID-19 pandemic, is uncertainty. Uncertainty is one of the key determinants of experienced levels of stress [28–30]. Moreover, the ability to deal with uncertainty varies widely. While some people can tolerate uncertainty very well, others have difficulties tolerating uncertainty and try to avoid it at best [31–33]. Intolerance of uncertainty (IU) is described as a predisposition to negatively perceive and respond to uncertain information and situations, irrespective of its probability and outcomes [34, 35]. As the worldwide COVID-19 pandemic influenced daily life for all people, escaping from the accompanied uncertainty is deemed impossible. Consequently, parents and adolescents with higher levels of IU might experience greater distress under the current circumstances, which might in turn also impact their affect and parenting behaviors. No prior studies have investigated the relation between IU and daily affect and parenting behavior within the family context. This was pursued in the present study. In the light of the pandemic, it is also examined to what extent IU is related to a change in affect and parenting behaviors.

### Present study

In the present study, we examined the impact of the COVID-19 pandemic on daily affect and parenting of both Dutch parents *and* adolescents. The aims were: (1) To explore parents’ and adolescents’ daily difficulties and helpful activities during the COVID-19 pandemic, (2) to examine and compare positive and negative affect of both parents and adolescents during 2 weeks of the COVID-19 pandemic and a similar 2-week period pre-pandemic (from now on referred to as baseline), (3) to examine and compare (perceived) parenting behaviors in terms of parental warmth and criticism towards the adolescent (as assessed by both the adolescent and the parent) during 2 weeks of the COVID-19 pandemic and a similar 2-week period pre-pandemic, (4) to examine whether parents’ and adolescents’ levels of IU at baseline are associated with affect and parenting behaviors in general, and (5) as well as with the hypothesized changes in affect and (perceived) parental warmth and criticism.

We expect an increase of negative affect and a decrease in positive affect for both parents and adolescents during the COVID-19 pandemic as compared to baseline. Regarding parenting behaviors, we expect lower levels of parental warmth and higher levels of parental criticism during the COVID-19 pandemic as compared to baseline, both from the perspective of parents and adolescents. With respect to IU, we expect that higher levels of IU predict higher levels of negative affect and lower levels of positive affect in parents and adolescents at both time points, as well as a greater increase in negative affect and decrease in positive affect during the COVID-19 pandemic compared to baseline.

## Method

### Sample

The current study was based on baseline data of the ongoing Dutch multi-method two-generation RE-PAIR study: ‘*Relations and Emotions in Parent-Adolescent Interaction Research*’ and on the follow-up assessment ‘RE-PAIR during the COVID-19 pandemic’. In RE-PAIR, we examine the relation between parent-child interactions and adolescent mental well-being. The study design and in- and exclusion criteria of the baseline assessment can be found in S1 Text. The current study included data from adolescents without psychopathology and their parents (i.e., healthy control families).

Inclusion criteria for the adolescents to participate in the current study at baseline were: being aged between 11 and 17 years, living at home with at least one primary caregiver, going to high school or higher education, and a good command of the Dutch language. Adolescents were excluded if they had a current mental disorder, a life-time history of major depressive disorder or dysthymia, or a history of psychopathology in the past two years. Adolescent psychopathology was assessed at baseline during a face-to-face interview using the Structured Interview of the Kiddie-Schedule for Affective Disorders and Schizophrenia—Present and Lifetime Version (K-SADS-PL [36]). For parents, no in- or exclusion criteria were specified, except for a good command of the Dutch language. To participate in the follow-up during the COVID-19 pandemic the adolescent had to still live at home with at least one caregiver. Adolescents and parents were allowed to sign up individually.

From the 80 adolescents and 151 parents who were contacted for the follow-up assessment during the COVID-19 pandemic, 51 individuals (14 adolescents and 37 parents) did not respond to any of the attempts of contact from the researchers. Of the individuals who did respond, 76 (31 adolescents and 45 parents) were not willing to participate. Reasons were: being busy and having other priorities (i.e., work, school, taking care of children or parents). The remaining 104 participants gave consent to participate. Two participants did not start the EMA and one participant did not complete the measures and hence, the final sample of the current study included 101 participants, consisting of 34 adolescents and 67 parents. Descriptive statistics of the current sample are described in the result section and in Table 1.

**Table 1:** Sample characteristics and study variables.

Variables	*N*	*Before COVID-19*	*During COVID-19*
**Parents**			
Gender, % Female, (*n)*	67	56.7 (38)	56.7 (38)
Age (years), *M (SD)*	67	48.23 (5.79)	49.12 (5.73)
Highest education ^a^, *% (n)*	67		
Lower vocational education		**3 (2)**	**3 (2)**
Intermediate vocational education		25.4 (17)	25.4 (17)
Higher vocational education or scientific education (university)		71.6 (48)	71.6 (48)
Depressive symptoms (PHQ-9), *M (SD)*	67	2.45(2.78)	2.87 (2.76)
Intolerance of Uncertainty (IUS), *M (SD)*	64	27.81 (6.51)	-
Positive affect ^a^, *M (SD)*	67	5.33 (0.65)	5.32 (0.73)
Negative affect ^a^, *M (SD)*		1.53 (.56)	1.65 (.62)
Parental warmth ^a^, *M (SD)*		5.64 (.70)	5.66 (.65)
Parental criticism ^a^, *M (SD)*		2.41 (1.01)	2.47 (1.02)
Adolescents			
Gender, % Girl *(n)*	34	64.7(22)	64.7(22)
Age (years), *M (SD)*	34	16.00 (1.15)	16.95 (1.01)
Current educational Level, *% (n)*	34		
Lower vocational education		5.9 (2)	5.9 (2)
Higher vocational education		32.4(11)	20.6 (7)
Pre-university education		50.0 (17)	50.0 (17)
Secondary vocational education		5.9 (2)	8.8 (3)
Higher vocational education		5.9 (2)	11.8 (4)
No current education		0.0 (0)	2.9 (1)
**Depressive symptoms (PHQ-9), *M (SD)***	34	4.21 (2.54)	4.82 (3.42)
Intolerance of Uncertainty (IUS), *M (SD)*	32	30.28 (6.59)	-
Positive affect ^a^	34	5.56 (.66)	5.54 (.75)
Negative affect ^a^	34	1.40 (.48)	1.44 (.47)
Parental warmth—mother ^a^	34	5.80 (.86)	5.70 (1.11)
Parental warmth—father ^a^	34	5.73 (1.14)	5.81 (1.11)
Parental criticism- mother ^a^	34	2.01 (.91)	2.15 (1.10)
Parental criticism- father ^a^	34	1.92 (.92)	1.97 (1.15)

^a^ person-mean.

### Procedure

Recruitment of the participants was done via social media, advertisements, and flyers, with a specific focus on the inclusion of *both* parents (i.e., mothers *and* fathers). The focus was on primary caregivers, so not only biological parents could participate, but also stepparents and guardians, as long as they played an important role in the upbringing of the adolescent. Interested families could sign-up for the study via the website or mail and received information letters. Approximately two weeks later families were contacted by phone by one of the researchers to provide them with more information and check the inclusion criteria. If all criteria were met, families could participate in the study. All participants signed informed consent (including consent to contact them to request to participate in follow-up research). In addition, for adolescents younger than 16 years of age, both parents with legal custody signed informed consent.

The families completed the EMA in the period between September 2018 and November 2019 with EMA not taking place during holidays and exam weeks of the adolescent. Instructions on the EMA were given face-to-face prior to the baseline assessment and researchers assisted with installing the Ethica app [37] on the smartphone of the adolescent and both parents. Each family member also received written instructions and their individual account information. For participation in the EMA, parents received €20,- and adolescents €10,-. In addition, four gift vouchers of €75,- were raffled based on compliance.

All families who participated at baseline were invited for the follow-up in April 2020. The follow-up assessment was announced in a newsletter followed by a personal e-mail, and reminders were sent to parents and adolescents who had not responded yet. Parents and adolescents who agreed to participate were sent an online questionnaire on demographic characteristics and general mental well-being. Thereafter, participants received written instructions on how to download and reinstall the Ethica app. EMA data collection took place one month into the lockdown, from April 14^th^ to April 28^th^. For participation in the follow-up assessment, parents received €20,- and adolescents €10,- in gift vouchers. The current study focusses on the EMA data of the baseline assessment (2018–2019) and the follow-up assessment (2020).

The RE-PAIR study was approved by the Medical Ethics Committee of Leiden University Medical Center (LUMC) in Leiden, the Netherlands (NL62502.058.17) and the follow-up assessment ‘RE-PAIR during the COVID-19 pandemic’ was approved by the Psychology Research Ethics Committee of Leiden University in Leiden, the Netherlands (2020-03-30-B.M. Elzinga-V2-2334).

#### EMA

The EMA procedures and set-ups were almost entirely similar at baseline and during the COVID-19 pandemic and consisted of filling out questionnaires at four timepoints per day, for 14 consecutive days on parents’ and adolescents’ own smartphones using the mobile app Ethica (Ethica Data, 2019). At all timepoints participants completed questions about their affect and how they experienced contact with the last person they interacted with. Detailed information on the concepts in the questionnaires, triggering schedules, differences in set-up, number of items and completing time, and monitoring process can be found in S2 Text.

#### Compliance

The overall response rate at baseline was 81.0%. Adolescents completed 74.2% of the EMA questionnaires at baseline (M = 41.56 completed, SD = 9.21, Min/Max = 12/54). Parents completed 84.1% of the EMA questionnaires at baseline (M = 47.12 completed, SD = 6.32, Min/Max = 29/56). The overall response rate during the COVID-19 pandemic was 72.1%. Adolescents completed 64.6% of the EMA questionnaires during the COVID-19 pandemic (M = 36.18 completed, SD = 13.71, Min/Max = 8/54). Parents completed 75.9% of the EMA questionnaires during the COVID-19 pandemic (M = 42.49 completed, SD = 9.17, Min/Max = 21/56). No participants were excluded based on EMA compliance.

### EMA measures

#### Affect

Momentary affect states of parents and adolescents were assessed four times per day with a slightly adapted and shortened four-item version of the Positive and Negative Affect Schedule for Children (PANAS-C; [38, 39]). At each timepoint participants were asked “How do you feel at the moment?” followed by two positive affect states “Happy” and “Relaxed”, and two negative affect states “Sad” and “Irritated”. Each affect state was rated on a 7-point Likert scale, ranging from 1 (*not at all*) to 7 (*very*). A mean score of the positive affect state was calculated per moment to create a momentary PA scale and a mean score of the negative affect state was calculated per moment to create a momentary NA scale. A higher score represented higher levels of PA or NA.

#### Daily parenting

In the last questionnaire of each day, adolescents were asked to indicate with whom they spoke during that day (i.e., mother, father, stepmother, stepfather), and if so, to rate each parent’s warmth and criticism by answering the questions “Throughout the day, how warm/loving was your parent towards you?” and “Throughout the day, how critical was your parent towards you?” on a 7-point Likert scale ranging from 1 (*not at all*) to 7 (*very*). If adolescents only reported on mother and stepfather for instance throughout the EMA, scores about stepfathers were recoded as father. This was the case for two adolescents during the baseline and three adolescents during the COVID-19 pandemic. One adolescent reported on four caregivers (i.e. biological parents and stepparents) during both periods and we included scores about biological parents because these were mostly rated.

In the questionnaire at the end of each day parents also had to indicate whether they spoke to their child (i.e., the participating adolescent) and if so, to rate their own behavior towards their child by answering the questions “How warm/loving were you towards your child?” and “How critical were you towards your child?” on a 7-point Likert scale ranging from 1 (*not at all*) to 7 (*very*). Both for adolescent and parent report, a higher score represented more warmth and more criticism.

#### Daily difficulties and helpful activities

To assess the difficulties and helpful activities during the COVID-19 pandemic, at the end of each day, participants were asked to choose items from a list of potential activities. Parents and adolescents could select almost similar activities and it was possible to give multiple answers. The list of potential daily difficulties consisted of: boredom, fights/conflicts, work (for parents)/homework (for adolescents), irritations with family members, noise disturbance, loneliness, missing social contact with friends, worries about own health, worries about health of others, concerns about the coronavirus in general, coronavirus-related news items or ‘anything else, namely…’. The list of potential helpful activities consisted of: work (for parents)/homework (for adolescents), watching series/television, listening to music, gaming, social media, reading a book, sports, chilling, online contact with relatives or friends, being together with the family, card or board games, DIY or crafts, cooking/dining, ‘anything else, namely’. Based on the total number of observed responses a top 5 of daily difficulties and helpful activities was composed. Percentages were calculated by dividing the number of observed responses on one activity by the total of given answers.

### Questionnaires

#### Intolerance of uncertainty

The 12-item version of the Intolerance of Uncertainty Scale (IUS; [40]) was used to assess IU of parents and adolescents. Participants completed this questionnaire online prior to baseline. The 12 items of the IUS (e.g., “Uncertainty makes me uneasy, anxious, or stressed.” or “I should be able to organize everything in advance.”) were answered on a 5-point Likert scale ranging from 1 (*strongly disagree*) to 5 (*strongly agree*). A higher sum score represents higher levels of intolerance of uncertainty. Both the original and the 12-item version of the IUS appear to have satisfactory concurrent, discriminant, and predictive validity [41]. Internal consistency of the scale was good with a Cronbach’s alpha of .81 for adolescents and .83 for parents.

#### Depressive symptoms

The Patient Health Questionnaire (PHQ-9; [42]) was used to screen for the presence of depressive symptoms during the past two weeks. Depressive symptoms were assessed at both timepoints. The items are based on nine DSM-IV criteria for depression and are scored as 0 (*not at all*) to 3 (*nearly every day*). The PHQ-9 has been validated for use in primary care. Sum scores range from 0 to 27 and a score above 10 is suggestive of the presence of depression [43]. For parents, the Cronbach’s alpha at baseline was .79 and during the COVID-19 pandemic .73. For adolescents, Cronbach’s alpha at baseline was .53 and during the COVID-19 pandemic .76.

### Strategy of analyses

Parents and adolescents reported repeatedly on positive affect, negative affect, parental warmth, and parental criticism at baseline and during the COVID-19 pandemic. These repeated measures (Level 1) were nested within individuals (Level 2). Given this nested structure of the data, multilevel modelling [44] was used for the main analyses. Models were specified in R Version 3.6.1 [45], using the multilevel version 2.6 [46] package to test our hypotheses with maximum likelihood (ML) estimation. Level 2 predictors were grand-mean centered, following guidelines proposed by Hoffman [47] and Bolger and Laurenceau [48].

To evaluate within-person change in positive affect, negative affect, parental warmth, and parental criticism from baseline to the COVID-19 pandemic, a series of models were tested. Separate models were tested per outcome and per informant (adolescents and parents), resulting in a total of 8 models. Per model, several similar steps were taken. First, we specified an unconditional random intercept model with covariance structure (Model 1). For more information on the selection of covariance structure and results see S3 Text. Second, we added period as predictor (Model 2), which was scored 0 (baseline) and 1 (during the COVID-19 pandemic) to model change. For example, to model change in positive affect, we specified period as the predictor and positive affect as the outcome. The intercept of the model estimates is positive affect score at baseline and the slope of the model is the estimated change from baseline to during the COVID-19 pandemic. Fourth, we added a random effect (Model 3) indicating that the change from baseline to during the COVID-19 pandemic could vary between persons. Significant changes in model fit were tested with likelihood ratio tests (following guidelines of Hox [44]). Fifth, we examined whether the changes were predicted by IU by adding a main effect of IU (Model 4). In the models on parental warmth and parental criticism gender of parents was also added to the model as main effect to test for possible gender differences. In the final model (Model 5), we also added an interaction term of IU with period to test the possible moderating role of IU.

Since two parents of a same family could participate in the study, a third level (family) was specified in all models including parents (Model 1b). To not overcomplicate our models, we tested whether adding family level (Level 3) to Model 1 for parents improved the model fit based on the likelihood ratio tests. Only if these tests were significant, the third level remained in the model. Since adolescents could report on parenting of fathers and mothers, family was specified as extra level in the models concerning parental warmth and parental criticism reported by adolescents (Model 1b). For adolescents, answers on father and mother (Level 2) are nested within adolescents (Level 3). We tested whether adding parent level (Level 2) to Model 1 for adolescents improved the model fit based on the likelihood ratio tests. If these tests were significant, the second level remained in the model.

We used two-tailed tests with an α = 0.05. The analytic plan for this study was uploaded to Open Science Framework prior to the analyses (preregistered at April 27^th^, osf.io/34ycu).

## Results

### Sample description

In the current study, 67 Dutch parents (age range during the COVID-19 pandemic: 36.25–71.04 years) and 34 adolescents (age range during the COVID-19 pandemic: 14.66–19.01 years) participated. Participant characteristics can be found in Table 1. The sample reported little to none depressive symptoms as measured with the PHQ-9. PHQ-9 scores of adolescents ranged between 0–9 at baseline and between 0–16 during the COVID-19 pandemic. PHQ-9 scores of parents ranged between 0–16 at baseline and between 0–16 during the COVID-19 pandemic. Levels of depressive symptoms did not differ between the two periods for adolescents (*t* = 1.11, *df* = 33, *p* = .275) and parents (*t* = 1.24, *df* = 67, *p* = .221). Information on household composition of participating families can be found in S3 Text. Correlations between study variables (gender, age, affect, parenting behavior, and IU) can be found in S1 Table (parents) and S2 Table (adolescents).

### Situational description of the families during the COVID-19 pandemic

#### Parents

Of all parents, 91% (*n* = 61) were currently employed, 6% (*n* = 4) were unemployed and 3% (*n* = 2) were unable to work or lost their job due to the COVID-19 pandemic. During the 14 days of EMA, 53.7% of the parents who were employed worked more from home, 7.5% worked less from home and 38.8% worked just as much from home as compared to the period before the COVID-19 pandemic. All parents indicated owning a house with a garden and having a living surface >100m2. Of our sample, 17.9% (*n* = 12) of the parents reported having COVID-19 related symptoms during the 14 days of EMA.

During the COVID-19 pandemic, the most reported daily difficulties across the 14 days of EMA for parents were (1) missing social contact with friends (14.6%), (2) concerns about the coronavirus in general (13.5%), (3) irritations with family members (12.8%), (4) worrying about health of others (8.3%), and (5) coronavirus-related news items (8.0%). It was also asked daily which activities were helpful during the day. The top 5 of helpful activities reported by parents was (1) being together with family (20.0%), (2) cooking/dining (14.4%), (3) watching television/series (9.9%), (4) work (7.4%), and (5) online contact with relatives or friends (6.2%).

#### Adolescents

Due to the COVID-19 pandemic all national final school exams were canceled and some high schoolers already graduated (or not) based on their prior school exams, 5 (21.7%) adolescents graduated promptly in March 2020 prior to the 14 days of EMA. Of our adolescent sample, one person reported having COVID-19 related symptoms during the 14 days of EMA.

For adolescents (*n* = 34) the top 5 daily difficulties was (1) boredom (22.9%), (2) missing social contact with friends (17.7%), (3) irritations with family members (13.1%), (4) homework (12.3%), and (5) worry about the health of others (6.4%). The top 5 helpful activities for adolescents were (1) chilling (12.9%), (2) watching television/series (11.4%), (3) online contact with relatives or friends (11.0%), (4) listening to music (10.8%), and (5) being together with the family (9.6%).

### Affect during the COVID-19 pandemic versus baseline

#### Affect: Parent reports

First, an unconditional means model of negative affect with the intercept only was built (referred to as ‘Model 1’- complete model results of parents can be found in S3 Table, model fit statistics of parents can be found in S4 Table). The intraclass correlation coefficient (ICC) was .31 on the person level, indicating that moderate concordance of negative affect across time points within persons existed. Next, family was added as level to the unconditional means model (Model 1b). The ICC of the family level was .11, which indicates that some concordance of negative affect existed within families. However, the model fit did not improve significantly (*χ2*(1) = 1.581, *p* = .209) and family level was therefore removed from the model.

**Fig 2:**
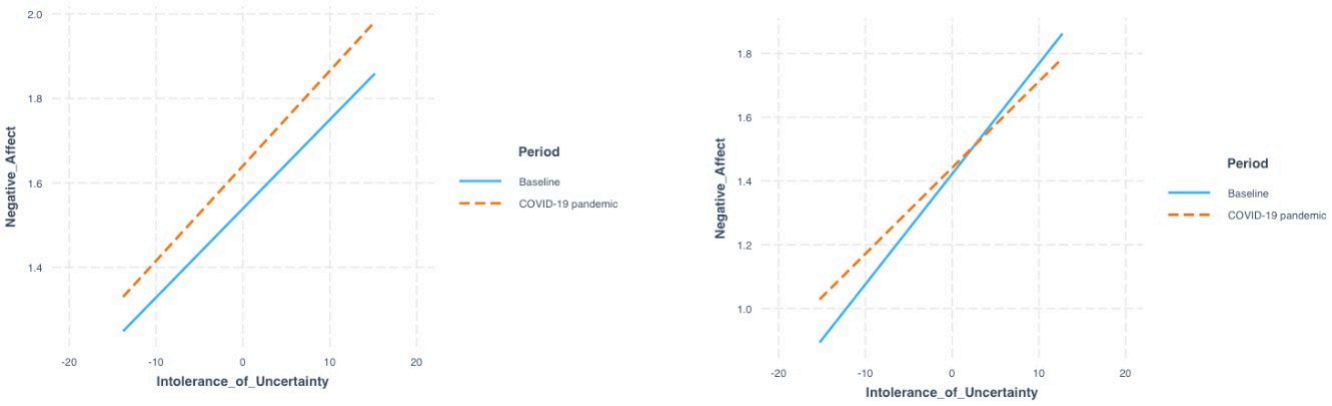
Association between negative affect and IU grouped per period for parents (left) and adolescents (right).

Next, in Model 2, we tested change in negative affect from baseline to during the COVID-19 pandemic by adding period to the model. Parents reported more negative affect during COVID-19 pandemic as compared to the baseline (*B* = 0.096, *SE* = .025, *df* = 5982, *t* = 3.900, *p* < .001). Adding individual variance in Model 3 improved the model fit significantly (*χ2*(2) = 56.613, *p* < .001). In Model 4, we added IU which was significantly associated with negative affect (*B* = 0.022, *SE* = .010, *df* = 62, *t* = 2.075, *p* = .042) indicating that more IU was related to more negative affect (main effect). Lastly, we added IU as moderator in Model 5 and results of this final model are presented in Table 2. No moderating effect of IU was found (*B* = 0.002, *SE* = .007, *df* = 5752, *t* = 0.225, *p* = .822) and IU was no longer significantly associated with negative affect (*B* = 0.021, *SE* = .011, *df* = 62, *t* = 1.960, *p* = .054), but period remained significantly associated with negative affect. Results are shown in Fig 2.

**Table 2:** Results of final model 5 on the relation between period and affect and the moderating role of intolerance of uncertainty in parents.

	Model 5: negative affect				Model 5: positive affect			
	*B*	*SE*	*t*	*p*	*B*	*SE*	*t*	*p*
Intercept	1.539	.069	22.224	< .001	5.321	.081	65.657	< .001
Period (baseline vs COVID-19)	0.105	.043	2.422	.016	-0.002	.060	-0.040	.968
IU	0.021	.011	1.960	.054	-0.015	.013	-1.177	.244
IU*Period	0.002	.007	0.225	.822	-0.008	.009	-0.823	.411
Random effects								
Between-person variance	0.288				0.397			
Within-person variance	0.635				0.768			
Random effect variance	0.082				0.182			
N parents	64				64			
N observations	5818				5822			

Note. 64 parents are included in these models since 3 parents did not complete the IUS.

For positive affect, the same steps were followed. Model 1 showed an ICC of .32 and adding family level (Model 1b) did not significantly improve the model fit (*χ2*(1) = 0.738, *p* = .390). Results of Model 2 showed that parents’ positive affect did not differ across the two periods (*B* = 0.012, *SE* = .028, *df* = 5986, *t* = 0.404, *p* = .686). Adding individual variance in Model 3 improved the model fit significantly (*χ2*(2) = 122.186, *p* < .001). In Model 4 IU was added as a main effect, but no significant association with positive affect was found. Lastly, IU was added as moderator in Model 5, but no moderating effect of IU was found (*B* = -0.008, *SE* = .009, *df* = 5756, *t* = -0.823, *p* = .411). Results of this final model are presented in Table 2.

#### Affect: Adolescent reports

In Model 1, the ICC of negative affect on the person level was .32 (complete model results of adolescents can be found in S5 Table, model fit statistics of adolescents can be found in S6 Table). Results of Model 2 showed that there was no significant change in adolescent negative affect (*B* = 0.016, *SE* = .027, *df* = 2618, *t* = 0.595, *p* = .552). Adding individual variance in Model 3 improved the model fit significantly (*χ2*(2) = 39.759, *p* < .001). In Model 4, we added IU as a main effect which was significantly associated with negative affect (*B* = 0.030, *SE* = .011, *df* = 30, *t* = 2.737, *p* = .010) indicating that more IU was related to more negative affect. IU was added as moderator in Model 5 and IU remained significantly associated with negative affect, but no moderating effect of IU was found (*B* = -0.006, *SE* = .008, *df* = 2463, *t* = -0.803, *p* = .422). Results of this final model are presented in Table 3. Results are shown in Fig 2.

**Table 3:** Results of final model 5 on the relation between period and affect and the moderating role of intolerance of uncertainty in adolescents.

	Model 5: negative affect				Model 5: positive affect			
	*B*	*SE*	*t*	*p*	*B*	*SE*	*t*	*p*
Intercept	1.419	.078	18.201	< .001	5.516	.106	52.223	< .001
Period (baseline vs COVID-19)	0.032	.052	0.626	.532	-0.008	.111	-0.075	.940
IU	0.034	.012	2.827	.008	-0.043	.016	-2.626	.014
IU*Period	-0.006	.008	-0.803	.422	-0.003	.017	-0.199	.842
Random effects								
Between-person variance	0.183				0.333			
Within-person variance	0.391				0.675			
Random effect variance	0.060				0.339			
N adolescents	32				32			
N observations	2497				2497			

Note. 32 adolescents are included in these models since 2 adolescents did not complete the IUS.

For positive affect in Model 1, the ICC on the person level was .33. No significant change in adolescent positive affect (*B* = 0.025, *SE* = .043, *df* = 2618, *t* = 0.574, *p* = .566) was found in Model 2. Adding individual variance in Model 3 improved the model fit significantly (*χ*2(2) = 103.798, *p* < .001). In Model 4, we added IU as main effect, which was significantly associated with positive affect (*B* = -0.044, *SE* = .015, *df* = 30, *t* = -2.917, *p* = .007), indicating that more IU was related to less positive affect. IU was added as moderator in Model 5, IU remained significantly associated with positive affect, but no moderating effect of IU was found (*B* = -0.003, *SE* = .017, *df* = 2463, *t* = -0.199, *p* = .842). Results of this final model are presented in Table 3.

#### Parenting: Parent reports

In Model 1, the ICC of parental criticism on the person level was .39 (complete model results of parents can be found in S3 Table, model fit statistics of parents can be found in S4 Table). Adding family level (Model 1b) did significantly improve the model fit (*χ2*(1) = 5.430, *p* = .020) with an ICC of .20 at the family level and ‘family’ remained in the model. Results of Model 2 showed that no difference in parental criticism between baseline and during the COVID-19 pandemic was found (*B* = 0.126, *SE* = .064, *df* = 1530, *t* = 1.963, *p* = .050). Adding individual variance in Model 3 improved the model fit significantly (*χ2*(4) = 39.527, *p* < .001). In Model 4, we added IU and gender of the parent as main effects. Both were not significantly associated with parental criticism. IU was added as moderator in Model 5, but no moderating effect of IU was found (*B* = -0.013, *SE* = .014, *df* = 1466, *t* = -0.944, *p* = .346). Results of this final model are presented in Table 4.

**Table 4:** Results of final model 5 on the relation between period and daily parenting behavior and the moderating role of intolerance of uncertainty in parents.

	Model 5: parental criticism				Model 5: parental warmth			
	*B*	*SE*	*t*	*p*	*B*	*SE*	*t*	*p*
Intercept	2.363	.165	14.313	< .001	5.588	.110	50.808	< .001
Period (baseline vs COVID-19)	0.131	.112	1.169	.243	0.027	.055	0.499	.618
Gender	0.113	.178	0.636	.530	0.064	.157	0.405	.687
IU	-0.004	.018	-0.250	.805	-0.019	.013	-1.419	.161
IU*Period	-0.013	.014	-0.944	.346	0.004	.008	0.489	.625
Random effects								
Between-person variance	0.455				0.429			
Within-person variance	1.146				0.428			
Random effect variance	0.141				0.104			
Family variance	0.462							
Random effect variance	0.238							
N families	37							
N parents	64				64			
N observations	1532				1532			

Note. 64 parents are included in these models since 3 parents did not complete the IUS.

For parental warmth in Model 1, the ICC on the person level was .46 and adding family level (Model 1b) did not significantly improve the model fit (*χ2*(1) = 0.761, *p* = .383). No significant change in parental warmth (*B* = 0.010, *SE* = .038, *df* = 1530, *t* = 0.255, *p* = .799) was found in Model 2. Adding individual variance in Model 3 improved the model fit significantly (*χ*2(2) = 22.499, *p* < .001). In Model 4, we added IU and gender of parent and both were not significantly associated with parental warmth. IU was added as moderator in Model 5, but no moderating effect of IU was found (*B* = 0.004, *SE* = .008, *df* = 1466, *t* = .489, *p* = .625). Results of this final model are presented in Table 4.

#### Parenting: Adolescent reports

In Model 1, the ICC of parental criticism on the person level was .45 (complete model results of adolescents can be found in S5 Table, model fit statistics of adolescents can be found in S6 Table). Adding family level (Model 1b) did not significantly improve the model fit (*χ2*(1) = 2.925, *p* = .087). Results of Model 2 showed that the change in reports on parental criticism between baseline and during the COVID-19 pandemic was not significant (*B* = 0.036, *SE* = .062, *df* = 1350, *t* = 0.576, *p* = .565). Adding individual variance in Model 3 improved the model fit significantly (*χ*2(2) = 53.931, *p* < .001). In Model 4, we added IU and gender of parent as main effects. Gender of parent was significantly associated with reports on parental criticism (*B* = -0.121, *SE* = .058, *df* = 1268, *t* = -2.099, *p* = .036), indicating that adolescents reported more parental criticism of mothers than fathers. IU was not significantly associated with parental criticism. IU was added as moderator in Model 5, but no moderating effect of IU was found (*B* = 0.028, *SE* = .021, *df* = 1267, *t* = 0.083, *p* = .934). Results of this final model are presented in Table 5. Gender of parents remained significantly associated with parental criticism.

**Table 5:** Results of final model 5 on the relation between period and daily parenting behavior and the moderating role of intolerance of uncertainty in adolescents.

	Model 5: parental criticism				Model 5: parental warmth			
	*B*	*SE*	*t*	*p*	*B*	*SE*	*t*	*p*
Intercept	2.043	0.158	12.970	< .001	5.710	.170	33.528	< .001
Period (baseline vs COVID-19)	0.120	0.137	0.878	.380	-0.038	.113	-0.334	.738
Gender parent	-0.121	0.058	-2.099	.036	0.014	.077	0.186	.854
IU	0.028	0.024	1.172	.251	-0.031	.026	-1.203	.238
IU*Period	0.002	0.021	0.083	.934	-0.010	.017	-0.594	.553
Random effects								
Between-person variance	0.714				0.789			
Within-person variance	0.765				0.503			
Random effect variance	0.476				0.310			
Parent variance					0.110			
Random effect variance					0.026			
N adolescents	32				32			
N parents					63			
N observations	1302				1302			

Note. 32 adolescents are included in these models since 2 adolescents did not complete the IUS.

For parental warmth in Model 1, the ICC on the person level was .60 and adding family level (Model 1b) did significantly improve the model fit (*χ2*(1) = 25.314, *p* < .001) with an ICC of .05 at the family level and family remained in the model. No significant change in parental warmth (*B* = 0.026, *SE* = .051, *df* = 1317, *t* = 0.500, *p* = .617) was found in Model 2. Adding individual variance in Model 3 improved the model fit significantly (*χ*2(4) = 74.831, *p* < .001). In Model 4, we added IU and gender of parent and both were not significantly associated with parental warmth. IU was added as moderator in Model 5, but no moderating effect of IU was found (*B* = 0.002, *SE* = .021, *df* = 1267, *t* = 0.083, *p* = .934). Results of this final model are presented in Table 5.

#### Post hoc analyses on increase in parents’ negative affect during the COVID-19 pandemic

As IU did not explain why parents reported more negative affect during COVID-19 pandemic as compared to the baseline, we did some post hoc analyses to examine whether characteristics related to the lockdown and the COVID-19 pandemic were associated with the increase of parents’ negative affect. Living surface, income, having suffered from COVID-19 symptoms, helping children with school at home, working from home, going to work, daily difficulties during the past two weeks of COVID-19, and working with COVID-19 patients were examined (see S7 and S8 Tables for description of the EMA items). None of these characteristics were related to the increase of parents’ negative affect during the COVID-19 pandemic as compared to the baseline (all *p*-values < .001).

## Discussion

In this study we (1) explored parents’ and adolescents’ daily difficulties and helpful activities during the COVID-19 pandemic (2) examined positive and negative affect of both parents and adolescents during 2 weeks of the COVID-19 pandemic and compared them to a 2-week baseline period pre-pandemic, (3) examined parenting behaviors (assessed by both the adolescent and the parent) and compared parental warmth and criticism towards the adolescent during 2 weeks of the COVID-19 pandemic and a 2-week baseline period, (4) examined whether parents’ and adolescents’ levels of IU at baseline are associated with affect and parenting in general, and (5) as well as with the hypothesized changes in affect and (perceived) parental warmth and criticism.

### Subjective experience of the COVID-19 pandemic

Most importantly, both parents and adolescents were bothered by a lack of social contact with friends, by irritations with family members, and worried about the health of others. This might be a logical consequence of the lockdown and social distancing. Remarkably, adolescents struggled with boredom whereas this was not the case for parents. Parents worried about the coronavirus in general, while this did not bother adolescents that much. In response to social distancing, online contact with relatives or friends aided both parents and adolescents to cope with the situation. In addition, watching tv-shows was also mentioned as a helpful activity by parents and adolescents. Other activities that helped to cope with the situation varied across parents and adolescents. While parents reported to benefit from being together with family and cooking and dining, adolescents reported chilling and listening to music.

### Negative affect

Previous studies have shown that quarantine and quarantine-related issues (i.e., financial insecurity, fear of infection, uncertainty about duration) in general have a negative influence on adult mood and mental well-being [9]. Therefore, it was expected that the COVID-19 pandemic and lockdown would increase negative affect and decrease positive affect as compared with a period before the lockdown. Our results show that, indeed, parents’ negative affect increased as compared to the period before the lockdown. Important to note is that we collected data during 5^th^ and 6^th^ week of the lockdown in the Netherlands with only minor prospects of easing regulations. We also explored whether other pandemic-related characteristics (i.e. living surface, income, relatives with COVID-19, hours of working at home, helping children with school and contact with COVID-19 patients at work) were linked to the increase of negative affect in parents. This was not the case.

Our findings suggested however the presence of heterogeneity among individuals. All our models improved significantly when allowing the associations between period (2 weeks of the COVID-19 pandemic versus a similar 2-week baseline period) and affect and parenting behavior to vary across individuals, which is in line with the theoretical notion of differential susceptibility (e.g., [49]). Whether or not parents and adolescents experience (emotional) problems during lockdown can clearly vary from household to household, suggesting that in general families seem to be able to adapt to the circumstances, but that some families struggle. This is important to keep in mind for potential future measures of social distancing.

It was expected that the forced social distance during the COVID-19 pandemic and particularly the physical distance from friends and peers and the school closure would result in an increase of negative affect and decrease of positive affect in adolescents (see also Loades et al. [50]). Yet, in our study, no differences in adolescent reports on negative affect were found during the COVID-19 pandemic as compared to a baseline period. As for adults, the opportunities for adolescents of online social interaction might have buffered feelings of isolation or loneliness and bolstered mental well-being during the COVID-19 pandemic [51]. Moreover, it should be noted that our sample is considered healthy on average, based on the PHQ-9 scores, and lived in relatively favorable circumstances (e.g., high socioeconomic status). Affect of adolescents with (subclinical) mental health issues (e.g. depressive or anxiety symptoms) or living under less fortune circumstances might be more influenced during the COVID-19 pandemic. Therefore, it is important to examine the effect of the COVID-19 pandemic in clinical samples to elucidate its effect on psychopathology. Moreover, it should be noted that our assessments were in the rather poignant phase of social lock down, when school closings may also have yielded relief for some adolescents. Even though individuals thrive to become independent during adolescence and start to explore the environment outside family household [2, 3] this period of enforced proximity did not seem to affect adolescents on the short-term. Potentially, the endurance of the lockdown may have more detrimental effects on adolescent well-being.

### Positive affect

Not for parents nor for adolescents, a change in positive affect was found. Despite the increase of stress and uncertainty around the COVID-19 pandemic, disasters such as a pandemic also might increase the sense of social connectedness and morality [10]. This sense of shared social identity and the feeling of ‘we are all in this together’ can be related to positive affect [20], which could explain why positive affect did not decrease in the present study. In families, as in our sample, no one was home alone, and one could still have online social interactions with others outside the household. To that end, ‘physical distancing’ might be a better term for the imposed social isolation or social distance, as was previously suggested in literature [10].

### Parenting

As mentioned before, the COVID-19 pandemic and the related lockdown may lead to more tension, irritability, and family conflicts or worse [10]. Notably, parent’ affect and parenting behavior are interrelated and are both involved in giving comfort, expressing approval or expressing criticism [52, 53]. For instance, parents who worry more, express more criticism towards their adolescents, indicating that a negative affect promotes insensitive and in more extreme cases abusive parenting behavior, whereas positive affect strongly relates to supportive parenting [52, 53]. Regarding parenting behaviors, we therefore expected higher levels of parental criticism and lower levels of parental warmth during the COVID-19 pandemic as compared to baseline. We found, however, that parental warmth and criticism from both parent and adolescent perspective, did not differ between before and during the COVID-19 pandemic. Interestingly, even though negative affect of parents increased compared to the period before lockdown, this did not seem to affect parenting behavior (self-report and perceived by the adolescent). It should be noted that, in general, adolescents perceived their mothers as more critical compared with fathers, unrelated to measurement period. This might be due to the unique roles of mothers and fathers in caregiving and setting rules and boundaries [54, 55].

### Intolerance of uncertainty (IU)

Results showed that IU was related to more negative affect in both parents and adolescents, independent of the period of assessment. Furthermore, in adolescents, IU was also linked to a decrease in positive affect, while for parents no link between IU and positive affect was found. It was expected that people with elevated IU levels might experience even greater distress under the COVID-19 circumstances as compared to baseline, however our results do not support this. IU is often described as a predisposition to negatively perceive and respond to uncertain information and situations, irrespective of its probability and outcomes [34, 35]. Apparently, it is negatively associated with affect in daily life, regardless of whether there are major threats and uncertainties, or more daily hassles. Future research could elucidate why IU may particularly dampen positive affect in adolescents and not in adults. Even though IU seems to relate to affect of parents and adolescents, it did not seem to spill over into parenting behaviors. These results give a first indication that IU also relates to more micro processes in daily life, for both adolescents and parents.

### Strengths, limitations and remarks

Firstly, the intensive longitudinal study design with multiple assessments per day enabled us to gain more fine-grained insights in affect and parenting behaviors in daily life and to consider individual differences. Secondly, assessment during two periods, before and during the COVID-19 pandemic, allowed us to detect changes due to the COVID-19 pandemic. Next to the strengths, it should be acknowledged that the sample (67 parents and 34 adolescents) was relatively small. Second, it should be noted that the study sample consisted of overall healthy, well-functioning parents and adolescents. That is, adolescents were screened at baseline and were excluded if they had a current mental disorder, a history of psychopathology in the past two years, or a lifetime history of major depressive disorder or dysthymia. Moreover, the PHQ-9 scores of adolescents and parents indicated few depressive symptoms. Therefore, findings might not be applicable to adolescents and parents with (sub)clinical mental health problems or at-risk populations (e.g. refugees, low socioeconomic status), since these groups might be at increased risk of problems such as loneliness, negative affect or negative parenting practices during the COVID-19 pandemic. Lastly, it should be noted that information on long-term consequences of lockdown during the COVID-19 pandemic is lacking.

Prior research has suggested that the impact of stress can be altered by mindsets and appraisals of stressful events [10, 56, 57]. These factors could possibly explain the individual variations we found. For instance, people with low expectations of the course of events might adapt relatively well to new situations and, therefore, experience little emotional problems. Moreover, adaptive mindsets about stressful events might increase positive emotions and reduce negative health symptoms [58]. Considering these factors in future studies might be useful to elucidate individual differences in risk and resilience.

## Conclusion

In our study parents, but not adolescents, showed an increase of negative affect in a two-week period (14–28 April 2020) during the COVID-19 pandemic compared with a similar two-week baseline period pre-pandemic. Positive affect and parenting behaviors ‘warmth’ and ‘criticism’ did not change. It can be concluded that, on average, parents and adolescents in our sample seem to deal fairly well with the circumstances. Individuals and families differed however to what extent the COVID-19 pandemic influenced their affect and (perspective of) parenting behavior. Living surface, income, having suffered from COVID-19 symptoms, helping children with school at home, working from home, going to work, difficulties during COVID-19, and working with COVID-19 patients did not explain the increase of parental negative affect.

Policy makers and mental health professionals working to prepare for potential disease outbreaks should be aware that the experience of being quarantined might affect individuals differently. Each parent and adolescent could therefore benefit from a different coping strategy, as ‘one size does not fit all’. Providing easily accessible and safe ways to increase online contact for all ages and layers of society, recommending to search for distraction such as listening to music or watching television, and helping to accept the uncertain situation are for instance potential coping strategies. In this way, individuals can find ways that suit their own personal needs in order to benefit their well-being in times of a lockdown and social distancing measures.

## Supporting information

S1 Text Detailed information RE-PAIR study. (DOCX)

S2 Text Detailed information on EMA: Concepts in the questionnaires, triggering schedules, differences in set-up, number of items and completing time, and monitoring process. (DOCX)

S3 Text Information on household composition of participating families. (DOCX)

S4 Text Detailed information on covariance structure. (DOCX)

S1 Table Correlations between study variables for parents. (DOCX)

S2 Table Correlations between study variables for adolescents. (DOCX)

S3 Table Model results parents. Table A: Model results on the relation between period and negative affect, and the moderating role of intolerance of uncertainty in parents. Table B: Model results on the relation between period and positive affect, and the moderating role of intolerance of uncertainty in parents. Table C: Model results on the relation between period and parental criticism, and the moderating role of intolerance of uncertainty in parents. Table D: Model results on the relation between period and parental warmth, and the moderating role of intolerance of uncertainty in parents.
(DOCX)

S4 Table Model fit statistics of all models of parents. (DOCX)

S5 Table Model results adolescents. Table A: Model results on the relation between period and negative affect, and the moderating role of intolerance of uncertainty in adolescents. Table B: Model results on the relation between period and positive affect, and the moderating role of intolerance of uncertainty in adolescents. Table C: Model results on the relation between period and parental criticism, and the moderating role of intolerance of uncertainty in adolescents. Table D: Model results on the relation between period and parental warmth, and the moderating role of intolerance of uncertainty in adolescents.
(DOCX)

S6 Table Model fit statistics of all models of adolescents. (DOCX)

S7 Table Overview of EMA items used in the current study. (DOCX)

S8 Table Overview of EMA items of the exit questionnaire used in the current study. (DOCX)

## Abbreviations

AIC: Akaike information criterion
B: Unstandardized beta
BIC: Bayesian information criterion
COVID-19: Coronavirus disease 2019
df: Degrees of freedom
EMA: Ecological momentary assessment
fMRI: Functional magnetic resonance imaging
ICC: Intra-class correlation coefficient
IU: Intolerance of uncertainty
IUS: Intolerance of uncertainty scale
K-SADS-PL: Kiddie-schedule for affective disorders and schizophrenia—present and lifetime version
LL: Log-likelihood
LUMC: Leiden university medical center
M: Mean
ML: Maximum likelihood
p: p-value
PANAS-C: Positive and negative affect schedule for children
PHQ-9: Patent health questionnaire
RE-PAIR: Relations and emotions in parent-adolescent interaction research
SD: Standard deviation
SE: Standard error
t: T-statistic
