# Supplementary material for: Does the COVID-19 pandemic impact parents’ and adolescents’ well-being? An EMA-study on daily affect and parenting
Source: PLoS One. 2020 Oct 16;15(10):e0240962. doi: 10.1371/journal.pone.0240962 (PMC7567366; doi:10.1371/journal.pone.0240962)
Supplement: S1 Text — (DOCX) [file pone.0240962.s001.docx]

**S1 Text. Detailed information RE-PAIR.**

In RE-PAIR, we examine the relation between parent-child interactions and adolescent depression by comparing families with an adolescent with current major depressive disorder or dysthymia to families with an adolescent without psychopathology. Inclusion criteria for the adolescents to participate in the RE-PAIR study were aged between 11 and 17 years, living at home with at least one primary caregiver, going to high school or higher education, and a good command of the Dutch language. For the healthy control families of RE-PAIR, adolescents were excluded if they had a current mental disorder, a life-time history of major depressive disorder or dysthymia, or a history of psychopathology in the past two years. Adolescent psychopathology was assessed using the Structured Interview of the Kiddie-Schedule for Affective Disorders and Schizophrenia – Present and Lifetime Version (K-SADS-PL [1]). For parents, no in- or exclusion criteria were specified, except for a good command of the Dutch language.

If all criteria were met, families could participate in the RE-PAIR study which consisted of four parts: online questionnaires, a research day at the laboratory, two weeks of EMA, and an fMRI-scan session with the adolescent and one parent.

References

1. Reichart CG, Wals M, Hillegers M. Vertaling K-SADS. HC Rümke Groep, Utr. 2000;
