# Supplementary material for: Does the COVID-19 pandemic impact parents’ and adolescents’ well-being? An EMA-study on daily affect and parenting
Source: PLoS One. 2020 Oct 16;15(10):e0240962. doi: 10.1371/journal.pone.0240962 (PMC7567366; doi:10.1371/journal.pone.0240962)
Supplement: S2 Text — (DOCX) [file pone.0240962.s002.docx]

**S2 Text. Detailed information on EMA procedure and set-up**

*Concepts of questionnaires*

The EMA consisted of four questionnaires at four time points per day: one in the morning, two during the day and one in the evening. At all time points participants completed questions about their affect and how they experienced contact with the last person they interacted with (see S11 Table for an overview of the items that were used in this study). The first questionnaire of each day additionally included questions about the quality of sleep and the last questionnaire of each day about self-image, parenting behaviors, activities, and substance use (e.g., coffee, cannabis) throughout the day. The last questionnaire of each day during the follow-up furthermore included questions related to the COVID-19 situation; such as health issues, being in quarantine, working from home, and being worried about the pandemic.

*Triggering schedule*

Participants received the questionnaires between 7AM and 9.30PM on weekdays and 9AM and 9.30PM on weekend days according to a standardized trigger schedule. At baseline, the first questionnaire of each day was sent at 7AM on weekdays and 9AM during weekend days and expired after 120 minutes. Two questionnaires during each day were sent at a random time point between 12AM and 1PM, and between 4PM and 7PM. Both expired after 60 minutes. The last questionnaire of each day was sent to adolescents at a random time point between 8.15PM and 8.45PM and to parents between 9PM and 9.30PM, both expired after 180 minutes. Additionally, participants could receive questionnaires based on the proximity of family members, but these questionnaires were not used in the current study.

For the follow-up, small changes were made to the trigger schedule since it was expected that day routines would be slightly different (i.e., school and work from home). The first questionnaire of each day expired after 300 minutes on weekdays and after 180 minutes on weekend days. The questionnaires during each day were sent at a random time point between 1AM and 3PM, and between 4PM and 7PM. No changes were made in the trigger setting for the last questionnaire of each day.

*Number of items and survey completion time*

The EMA questionnaires at baseline consisted of minimal 14 items, 13 closed and 1 open, and maximal 45 items, 44 closed and 1 open. The EMA questionnaires at follow-up consisted of minimal 14 items, 13 closed and 1 open, and maximal 59 items, 58 closed and 1 open. Number of items depended on role (parent or adolescent), branching, and type of questionnaire (morning, day, or evening). On average filling out the questionnaires took participants 2.48 minutes (SD = 2.26) at baseline and 3.00 minutes (SD = 3.39) at follow-up.

*Monitoring process*

The EMA monitoring process at baseline and during the COVID-19 pandemic was similar. Researchers monitored the EMA by checking daily whether participants received and completed questionnaires and were available for questions via WhatsApp or phone. On day four, seven, and eleven an update was sent to each participant about the personal adherence (percentage) as motivation. On the last day of the EMA all participants received a message to thank them for participation. During the COVID-19 pandemic, participants were asked to complete an exit questionnaire the day after the last day of the EMA, including some general questions about the past two weeks (see S12 Table for an overview of the items that were used in questionnaire).
