# Supplementary material for: Does the COVID-19 pandemic impact parents’ and adolescents’ well-being? An EMA-study on daily affect and parenting
Source: PLoS One. 2020 Oct 16;15(10):e0240962. doi: 10.1371/journal.pone.0240962 (PMC7567366; doi:10.1371/journal.pone.0240962)
Supplement: S3 Text — (DOCX) [file pone.0240962.s003.docx]

**S3 Text. Information on household composition of participating families**

Of the families from which both parents and adolescents participated (N = 32), 1.6% (n = 1) consisted of a single parent household with one adolescent, 3.3% (n = 2) consisted of a single-parent household with two or more children, 13.1% (n = 8) consisted of a two-parent household with one adolescent, 77.0% (n = 47) consisted of a two-parent household with two or more children and 4.9% (n = 3) of the families consisted of a blended family household (e.g. multiple step children, switching between father and mother). Of the six parents who participated without an adolescent, four parents were part of a family consisting of a two-parent household with two or more children and two families consisting of a two-parent household with one child. Of the two adolescents who participated without a parent, one was a child of a two-parent household with at least two children and the other adolescent was a member of a blended family household.
