# Supplementary material for: Does the COVID-19 pandemic impact parents’ and adolescents’ well-being? An EMA-study on daily affect and parenting
Source: PLoS One. 2020 Oct 16;15(10):e0240962. doi: 10.1371/journal.pone.0240962 (PMC7567366; doi:10.1371/journal.pone.0240962)
Supplement: S4 Text — (DOCX) [file pone.0240962.s004.docx]

**S4 Text. Detailed information on covariance structure.**

We added a covariance structure to account for the time intervals between the observations [1]. We tested both corAR1 (equally spaced time intervals) and corCAR1 (unequally spaced time intervals) since we both used momentary assessments (unequally spaced) and daily assessments (equally spaced). Likelihood ratio tests were used to assess differences in fit of the models (following guidelines of Hox [2]).

In all models, model fit improved significantly when adding covariance structure corAR1 and corCAR1 to the unconditional means model, but little differences in model fit were found between corAR1 and corCAR1. Therefore, corAR1 was used as covariance structure (model fit statistics can be found in S8 and S10 Table).

References

1. Singer JD, Willett JB. Applied longitudinal data analysis: Modeling change and event occurrence. Applied Longitudinal Data Analysis: Modeling Change and Event Occurrence. 2009. 1–644 p.
2. Hox J, Moerbeek M, Schoot R Van de. Multilevel analysis: Techniques and applications. 2010.
