# Supplementary material for: Does the COVID-19 pandemic impact parents’ and adolescents’ well-being? An EMA-study on daily affect and parenting
Source: PLoS One. 2020 Oct 16;15(10):e0240962. doi: 10.1371/journal.pone.0240962 (PMC7567366; doi:10.1371/journal.pone.0240962)
Supplement: S1 Table — (DOCX) [file pone.0240962.s005.docx]

**S1 Table. Correlations between study variables for parents (*n =* 67).**

|  |  | 1 | 2 | 3 | 4 | 5 | 6 | 7 | 8 | 9 | 10 | 11 | 12 |
| --- | --- | --- | --- | --- | --- | --- | --- | --- | --- | --- | --- | --- | --- |
| 1. Gender |  |  |  |  |  |  |  |  |  |  |  |  |  |
| 2. Age baseline |  | -.186 |  |  |  |  |  |  |  |  |  |  |  |
| 3. Age COVID-19 |  | -.167 | .966*** |  |  |  |  |  |  |  |  |  |  |
| 4. Person mean positive affect baseline |  | -.074 | -.020 | -.047 |  |  |  |  |  |  |  |  |  |
| 5. Person mean negative affect baseline |  | .097 | -.111 | -.088 | -.660*** |  |  |  |  |  |  |  |  |
| 6. Person mean positive affect COVID-19 |  | -.130 | .061 | .024 | .754*** | -.539*** |  |  |  |  |  |  |  |
| 7. Person mean negative affect COVID-19 |  | .104 | -.131 | -.103 | -.689*** | .821*** | -.723*** |  |  |  |  |  |  |
| 8. Person mean parental warmth baseline |  | .092 | .133 | .111 | .578*** | -.376** | .451*** | -.420*** |  |  |  |  |  |
| 9. Person mean parental criticism baseline |  | -.031 | -.037 | -.013 | -.367** | .494*** | -.266* | .436*** | -.509** |  |  |  |  |
| 10. Person mean parental warmth COVID-19 |  | -.070 | .147 | .118 | .593*** | -.442*** | .610*** | .493*** | .794*** | -.406*** |  |  |  |
| 11. Person mean parental criticism COVID-19 |  | -.004 | .020 | .051 | -.301* | .351** | .268* | .355** | -.428*** | .709*** | -.555*** |  |  |
| 12. Intolerance of uncertainty |  | .067 | -.179 | -.173 | -.150 | .241 | -.198 | .239 | -.183 | -.084 | -.162 | .153 |  |

**p* < .05. ***p* < .01. ****p* < .001.
