# Supplementary material for: Does the COVID-19 pandemic impact parents’ and adolescents’ well-being? An EMA-study on daily affect and parenting
Source: PLoS One. 2020 Oct 16;15(10):e0240962. doi: 10.1371/journal.pone.0240962 (PMC7567366; doi:10.1371/journal.pone.0240962)
Supplement: S2 Table — (DOCX) [file pone.0240962.s006.docx]

**S2 Table. Correlations between study variables for adolescents (*n =* 34).**

|  |  | 1 | 2 | 3 | 4 | 5 | 6 | 7 | 8 | 9 | 10 | 11 | 12 | 13 | 14 | 15 | 16 |
| --- | --- | --- | --- | --- | --- | --- | --- | --- | --- | --- | --- | --- | --- | --- | --- | --- | --- |
| 1. Gender |  |  |  |  |  |  |  |  |  |  |  |  |  |  |  |  |  |
| 2. Age baseline |  | -.028 |  |  |  |  |  |  |  |  |  |  |  |  |  |  |  |
| 3. Age COVID-19 |  | -.045 | .967*** |  |  |  |  |  |  |  |  |  |  |  |  |  |  |
| 4. Person mean positive affect baseline |  | -.163 | -.331 | -.330 |  |  |  |  |  |  |  |  |  |  |  |  |  |
| 5. Person mean negative affect baseline |  | .214 | -.134 | -.125 | -.654*** |  |  |  |  |  |  |  |  |  |  |  |  |
| 6. Person mean positive affect COVID-19 |  | -.275 | -.152 | -.150 | .617** | -.507** |  |  |  |  |  |  |  |  |  |  |  |
| 7. Person mean negative affect COVID-19 |  | .179 | -.142 | -.112 | -.470** | .796*** | -.608*** |  |  |  |  |  |  |  |  |  |  |
| 8. Person mean parental warmth mother baseline |  | .140 | -.303 | -.319 | .478** | -.263 | .412* | -.322 |  |  |  |  |  |  |  |  |  |
| 9. Person mean parental criticism mother baseline |  | -.104 | .144 | .186 | -.342* | .289 | .210 | .216 | -.586*** |  |  |  |  |  |  |  |  |
| 10. Person mean parental warmth father baseline |  | .126 | -.287 | -.262 | .468** | -.221 | .297 | -.110 | .862*** | -.489** |  |  |  |  |  |  |  |
| 11. Person mean parental criticism father baseline |  | -.270 | -.012 | -.002 | -.396* | .325 | -.129 | .086 | -.445* | .851*** | -.514** |  |  |  |  |  |  |
| 12. Person mean parental warmth mother COVID-19 |  | .077 | -.243 | -.214 | .324 | -.254 | .410* | .-.329 | .775*** | -.459** | .679*** | -.377* |  |  |  |  |  |
| 13. Person mean parental criticism mother COVID-19 |  | -.256 | .176 | .185 | -.317 | .171 | -.093 | .277 | -.569*** | .617*** | -.483** | .630*** | -.572*** |  |  |  |  |
| 14. Person mean parental warmth father COVID-19 |  | -.220 | .263 | .239 | -.519** | .461** | -.292 | .459** | -.628*** | .581*** | -.514** | .596*** | -.534** | .831*** |  |  |  |
| 15. Person mean parental criticism father COVID-19 |  | .060 | -.309 | -.294 | .457** | -.205 | .370* | -.115 | .779*** | -.462** | .810*** | -.465** | .888*** | -.486** | -.435* |  |  |
| 16. Intolerance of uncertainty |  | .326 | .094 | .070 | -.422* | .444* | -.395* | .390* | -.210 | .266 | -.177 | .133 | -.323 | .135 | .233 | -.172 |  |

**p* < .05. ***p* < .01. ****p* < .001.
