# Supplementary material for: Does the COVID-19 pandemic impact parents’ and adolescents’ well-being? An EMA-study on daily affect and parenting
Source: PLoS One. 2020 Oct 16;15(10):e0240962. doi: 10.1371/journal.pone.0240962 (PMC7567366; doi:10.1371/journal.pone.0240962)
Supplement: S3 Table — Table A: Model results on the relation between period and negative affect, and the moderating role of intolerance of uncertainty in parents. Table B: Model results on the relation between period and positive affect, and the moderating role of intolerance of uncertainty in parents. Table C: Model results on the relation between period and parental criticism, and the moderating role of intolerance of uncertainty in parents. Table D: Model results on the relation between period and parental warmth, and the moderating role of intolerance of uncertainty in parents. (DOCX) [file pone.0240962.s007.docx]

**S3 Table. Model results parents.**

**Table A. Results of Model 1, Model 1b, Model 2, Model3, and Model 4 on the Relation Between Period and Negative Affect and the Moderating Role of Intolerance of Uncertainty in Parents.**

|  | Model 1 | | | |  | Model 1b | | | |  | Model 2 | | | |  | Model 3 | | | |  | | Model 4 | | | | |
| --- | --- | --- | --- | --- | --- | --- | --- | --- | --- | --- | --- | --- | --- | --- | --- | --- | --- | --- | --- | --- | --- | --- | --- | --- | --- | --- |
|  | *B* | *SE* | *T* | *p* |  | *B* | *SE* | *t* | *p* |  | *B* | *SE* | *t* | *p* |  | *B* | *SE* | *t* | *p* |  | *B* | | *SE* | *t* | *p* |  |
| Intercept | 1.571 | .067 | 23.480 | < .001 |  | 1.586 | .076 | 20.831 | < .001 |  | 1.526 | .068 | 22.424 | <. 001 |  | 1.525 | .069 | 22.210 | < .001 |  | 1.539 | | .069 | 22.224 | < .001 |  |
| Period (baseline vs COVID-19) |  |  |  |  |  |  |  |  |  |  | 0.096 | .025 | 3.900 | < .001 |  | 0.101 | .042 | 2.413 | 0.016 |  | 0.105 | | .043 | 2.422 | .016 |  |
| IU |  |  |  |  |  |  |  |  |  |  |  |  |  |  |  |  |  |  |  |  | 0.022 | | .010 | 2.075 | .042 |  |
| Random effects |  |  |  |  |  |  |  |  |  |  |  |  |  |  |  |  |  |  |  |  |  | |  |  |  |  |
| Between-person variance | 0.289 |  |  |  |  | 0.199 |  |  |  |  | 0.291 |  |  |  |  | 0.298 |  |  |  |  | 0.288 | |  |  |  |  |
| Within-person variance | 0.644 |  |  |  |  | 0.644 |  |  |  |  | 0.641 |  |  |  |  | 0.621 |  |  |  |  | 0.635 | |  |  |  |  |
| Random effect variance |  |  |  |  |  |  |  |  |  |  |  |  |  |  |  | 0.079 |  |  |  |  | 0.082 | |  |  |  |  |
| ICC individual | 0.310 |  |  |  |  | 0.211 |  |  |  |  |  |  |  |  |  |  |  |  |  |  |  | |  |  |  |  |
|  |  |  |  |  |  |  |  |  |  |  |  |  |  |  |  |  |  |  |  |  |  | |  |  |  |  |
| Family variance |  |  |  |  |  | 0.099 |  |  |  |  |  |  |  |  |  |  |  |  |  |  |  | |  |  |  |  |
| ICC Family |  |  |  |  |  | 0.106 |  |  |  |  |  |  |  |  |  |  |  |  |  |  |  | |  |  |  |  |
|  |  |  |  |  |  |  |  |  |  |  |  |  |  |  |  |  |  |  |  |  |  | |  |  |  |  |
| N parents | 67 |  |  |  |  | 67 |  |  |  |  | 67 |  |  |  |  | 67 |  |  |  |  | 64 | |  |  |  |  |
| N observations | 6050 |  |  |  |  | 6050 |  |  |  |  | 6050 |  |  |  |  | 6050 |  |  |  |  | 5818 | |  |  |  |  |

**Table B. Results of Model 1, Model 1b, Model 2, Model3, and Model 4 on the Relation Between Period and Positive Affect and the Moderating Role of Intolerance of Uncertainty in Parents.**

|  | Model 1 | | | |  | Model 1b | | | |  | Model 2 | | | |  | Model 3 | | | |  | | Model 4 | | | | |
| --- | --- | --- | --- | --- | --- | --- | --- | --- | --- | --- | --- | --- | --- | --- | --- | --- | --- | --- | --- | --- | --- | --- | --- | --- | --- | --- |
|  | *B* | *SE* | *T* | *p* |  | *B* | *SE* | *t* | *p* |  | *B* | *SE* | *t* | *p* |  | *B* | *SE* | *t* | *p* |  | *B* | | *SE* | *t* | *p* |  |
| Intercept | 5.345 | .077 | 69.679 | < .001 |  | 5.343 | .082 | 64.956 | < .001 |  | 5.340 | .078 | 68.565 | < .001 |  | 5.342 | .079 | 67.492 | < .001 |  | 5.321 | | .081 | 65.629 | < .001 |  |
| Period (baseline vs COVID-19) |  |  |  |  |  |  |  |  |  |  | 0.012 | .028 | 0.404 | .686 |  | -0.002 | .058 | -0.037 | .970 |  | -0.002 | | .060 | -0.041 | .967 |  |
| IU |  |  |  |  |  |  |  |  |  |  |  |  |  |  |  |  |  |  |  |  | -0.017 | | .012 | -1.442 | .154 |  |
| Random effects |  |  |  |  |  |  |  |  |  |  |  |  |  |  |  |  |  |  |  |  |  | |  |  |  |  |
| Between-person variance | 0.380 |  |  |  |  | 0.312 |  |  |  |  | 0.380 |  |  |  |  | 0.397 |  |  |  |  | 0.397 | |  |  |  |  |
| Within-person variance | 0.795 |  |  |  |  | 0.795 |  |  |  |  | 0.794 |  |  |  |  | 0.750 |  |  |  |  | 0.768 | |  |  |  |  |
| Random effect variance |  |  |  |  |  |  |  |  |  |  |  |  |  |  |  | 0.177 |  |  |  |  | 0.185 | |  |  |  |  |
| ICC individual | 0.324 |  |  |  |  | 0.265 |  |  |  |  |  |  |  |  |  |  |  |  |  |  |  | |  |  |  |  |
|  |  |  |  |  |  |  |  |  |  |  |  |  |  |  |  |  |  |  |  |  |  | |  |  |  |  |
| Family variance |  |  |  |  |  | 0.070 |  |  |  |  |  |  |  |  |  |  |  |  |  |  |  | |  |  |  |  |
| ICC Family |  |  |  |  |  | 0.060 |  |  |  |  |  |  |  |  |  |  |  |  |  |  |  | |  |  |  |  |
|  |  |  |  |  |  |  |  |  |  |  |  |  |  |  |  |  |  |  |  |  |  | |  |  |  |  |
| N parents | 67 |  |  |  |  | 67 |  |  |  |  | 67 |  |  |  |  | 67 |  |  |  |  | 64 | |  |  |  |  |
| N observations | 6054 |  |  |  |  | 6050 |  |  |  |  | 6054 |  |  |  |  | 6054 |  |  |  |  | 5822 | |  |  |  |  |

**Table C. Results of Model 1, Model 1b, Model 2, Model3, and Model 4 on the Relation Between Period and Parental Criticism and the Moderating Role of Intolerance of Uncertainty in Parents.**

|  | Model 1 | | | |  | Model 1b | | | |  | Model 2 | | | |  | Model 3 | | | |  | | Model 4 | | | | |
| --- | --- | --- | --- | --- | --- | --- | --- | --- | --- | --- | --- | --- | --- | --- | --- | --- | --- | --- | --- | --- | --- | --- | --- | --- | --- | --- |
|  | *B* | *SE* | *T* | *p* |  | *B* | *SE* | *t* | *p* |  | *B* | *SE* | *t* | *p* |  | *B* | *SE* | *t* | *p* |  | *B* | | *SE* | *t* | *p* |  |
| Intercept | 2.427 | .114 | 21.299 | < .001 |  | 2.462 | .013 | 18.281 | < .001 |  | 2.402 | .139 | 17.315 | < .001 |  | 2.402 | .140 | 17.211 | < .001 |  | 2.363 | | .164 | 14.370 | < .001 |  |
| Period (baseline vs COVID-19) |  |  |  |  |  |  |  |  |  |  | 0.126 | .064 | 1.963 | .050 |  | 0.115 | .108 | 1.065 | .287 |  | 0.129 | | .112 | 1.149 | .251 |  |
| Gender |  |  |  |  |  |  |  |  |  |  |  |  |  |  |  |  |  |  |  |  | 0.112 | | .178 | 0.630 | .534 |  |
| IU |  |  |  |  |  |  |  |  |  |  |  |  |  |  |  |  |  |  |  |  | -0.011 | | .017 | -0.660 | .515 |  |
| Random effects |  |  |  |  |  |  |  |  |  |  |  |  |  |  |  |  |  |  |  |  |  | |  |  |  |  |
| Between-person variance | 0.797 |  |  |  |  | 0.404 |  |  |  |  | 0.403 |  |  |  |  | 0.463 |  |  |  |  | 0.463 | |  |  |  |  |
| Within-person variance | 1.262 |  |  |  |  | 1.262 |  |  |  |  | 1.257 |  |  |  |  | 1.151 |  |  |  |  | 1.145 | |  |  |  |  |
| Random effect variance |  |  |  |  |  |  |  |  |  |  |  |  |  |  |  | 0.136 |  |  |  |  | 0.150 | |  |  |  |  |
| ICC individual | 0.387 |  |  |  |  | 0.194 |  |  |  |  |  |  |  |  |  |  |  |  |  |  |  | |  |  |  |  |
|  |  |  |  |  |  |  |  |  |  |  |  |  |  |  |  |  |  |  |  |  |  | |  |  |  |  |
| Family variance |  |  |  |  |  | 0.414 |  |  |  |  | 0.421 |  |  |  |  | 0.410 |  |  |  |  | 0.450 | |  |  |  |  |
| Random effect variance |  |  |  |  |  |  |  |  |  |  |  |  |  |  |  | 0.232 |  |  |  |  | 0.239 | |  |  |  |  |
| ICC Family |  |  |  |  |  | 0.199 |  |  |  |  |  |  |  |  |  |  |  |  |  |  |  | |  |  |  |  |
|  |  |  |  |  |  |  |  |  |  |  |  |  |  |  |  |  |  |  |  |  |  | |  |  |  |  |
| N families |  |  |  |  |  | 39 |  |  |  |  | 39 |  |  |  |  | 39 |  |  |  |  | 37 | |  |  |  |  |
| N parents | 67 |  |  |  |  | 67 |  |  |  |  | 67 |  |  |  |  | 67 |  |  |  |  | 64 | |  |  |  |  |
| N observations | 1598 |  |  |  |  | 1598 |  |  |  |  | 1598 |  |  |  |  | 1598 |  |  |  |  | 1532 | |  |  |  |  |

**Table D. Results of Model 1, Model 1b, Model 2, Model3, and Model 4 on the Relation Between Period and Parental Warmth and the Moderating Role of Intolerance of Uncertainty in Parents.**

|  | Model 1 | | | |  | Model 1b | | | |  | Model 2 | | | |  | Model 3 | | | |  | | Model 4 | | | | |
| --- | --- | --- | --- | --- | --- | --- | --- | --- | --- | --- | --- | --- | --- | --- | --- | --- | --- | --- | --- | --- | --- | --- | --- | --- | --- | --- |
|  | *B* | *SE* | *T* | *p* |  | *B* | *SE* | *t* | *p* |  | *B* | *SE* | *t* | *p* |  | *B* | *SE* | *t* | *p* |  | *B* | | *SE* | *t* | *p* |  |
| Intercept | 5.635 | .078 | 71.897 | < .001 |  | 5.630 | .084 | 67.094 | < .001 |  | 5.630 | .081 | 69.906 | < .001 |  | 5.627 | .085 | 65.886 | < .001 |  | 5.588 | | .110 | 50.820 | < .001 |  |
| Period (baseline vs COVID-19) |  |  |  |  |  |  |  |  |  |  | 0.010 | .038 | 0.255 | .799 |  | 0.018 | .053 | 0.343 | .732 |  | 0.027 | | .055 | 0.502 | .616 |  |
| Gender |  |  |  |  |  |  |  |  |  |  |  |  |  |  |  |  |  |  |  |  | 0.064 | | .157 | 0.408 | .685 |  |
| IU |  |  |  |  |  |  |  |  |  |  |  |  |  |  |  |  |  |  |  |  | -0.016 | | .012 | -1.340 | .185 |  |
| Random effects |  |  |  |  |  |  |  |  |  |  |  |  |  |  |  |  |  |  |  |  |  | |  |  |  |  |
| Between-person variance | 0.386 |  |  |  |  | 0.313 |  |  |  |  | 0.386 |  |  |  |  | 0.446 |  |  |  |  | 0.430 | |  |  |  |  |
| Within-person variance | 0.451 |  |  |  |  | 0.451 |  |  |  |  | 0.451 |  |  |  |  | 0.423 |  |  |  |  | 0.428 | |  |  |  |  |
| Random effect variance |  |  |  |  |  |  |  |  |  |  |  |  |  |  |  | 0.100 |  |  |  |  | 0.105 | |  |  |  |  |
| ICC individual | 0.461 |  |  |  |  | 0.374 |  |  |  |  |  |  |  |  |  |  |  |  |  |  |  | |  |  |  |  |
|  |  |  |  |  |  |  |  |  |  |  |  |  |  |  |  |  |  |  |  |  |  | |  |  |  |  |
| Family variance |  |  |  |  |  | 0.074 |  |  |  |  |  |  |  |  |  |  |  |  |  |  |  | |  |  |  |  |
| Random effect variance |  |  |  |  |  |  |  |  |  |  |  |  |  |  |  |  |  |  |  |  |  | |  |  |  |  |
| ICC Family |  |  |  |  |  | 0.088 |  |  |  |  |  |  |  |  |  |  |  |  |  |  |  | |  |  |  |  |
|  |  |  |  |  |  |  |  |  |  |  |  |  |  |  |  |  |  |  |  |  |  | |  |  |  |  |
| N families |  |  |  |  |  |  |  |  |  |  |  |  |  |  |  |  |  |  |  |  |  | |  |  |  |  |
| N parents | 67 |  |  |  |  | 67 |  |  |  |  | 67 |  |  |  |  | 67 |  |  |  |  | 64 | |  |  |  |  |
| N observations | 1598 |  |  |  |  | 1598 |  |  |  |  | 1598 |  |  |  |  | 1598 |  |  |  |  | 1532 | |  |  |  |  |
