# Supplementary material for: Does the COVID-19 pandemic impact parents’ and adolescents’ well-being? An EMA-study on daily affect and parenting
Source: PLoS One. 2020 Oct 16;15(10):e0240962. doi: 10.1371/journal.pone.0240962 (PMC7567366; doi:10.1371/journal.pone.0240962)
Supplement: S4 Table — (DOCX) [file pone.0240962.s008.docx]

**S4 Table. Model fit statistics of all models of parents.**

|  |  |  |  |  | ΔLL test | | | |
| --- | --- | --- | --- | --- | --- | --- | --- | --- |
| Title | Observations | LL | AIC | BIC | Comparison | Δ-2LL | *df* | *p* |
| Negative affect |  |  |  |  |  |  |  |  |
| a. Unconditional model | 6050 | -7362.004 | 14730.010 | 14750.130 |  |  |  |  |
| b. Unconditional model AR individual (Model 1) | 6050 | -7260.661 | 14529.320 | 14556.150 | a vs b | 202.685 | 1 | < .001 |
| c. Unconditional model CAR individual | 6050 | -7260.662 | 14529.320 | 14556.150 |  |  |  |  |
| d. Unconditional model AR individual and family (Model 1b) | 6050 | -7259.871 | 14529.740 | 14563.280 | b vs d | 1.581 | 1 | .209 |
| e. Period model (Model 2) | 6050 | -7253.095 | 14516.190 | 14549.730 |  |  |  |  |
| f. Period and random slope model (Model 3) | 6050 | -7224.789 | 14463.580 | 14510.530 | e vs f | 56.613 | 2 | < .001 |
| g. Period, random slope, and IU model (Model 4) | 5818 | -7008.304 | 14032.610 | 14085.960 |  |  |  |  |
| h. Period, random slope, IU, and interaction model (Model 5) | 5818 | -7008.729 | 14034.560 | 14094.580 |  |  |  |  |
|  |  |  |  |  |  |  |  |  |
| Positive affect |  |  |  |  |  |  |  |  |
| a. Unconditional model | 6054 | -8002.238 | 16010.480 | 16030.600 |  |  |  |  |
| b. Unconditional model AR individual (Model 1) | 6054 | -7855.855 | 15719.170 | 15746.540 | a vs b | 292.766 | 1 | < .001 |
| c. Unconditional model CAR individual | 6054 | -7855.855 | 15719.710 | 15746.540 |  |  |  |  |
| d. Unconditional model AR individual and family (Model 1b) | 6054 | -7855.486 | 15720.970 | 15754.510 | b vs d | 0.738 | 1 | .390 |
| e. Period model (Model 2) | 6054 | -7855.773 | 15721.550 | 15755.090 |  |  |  |  |
| f. Period and random slope model (Model 3) | 6054 | -7794.680 | 15603.360 | 15650.320 | e vs f | 122.186 | 2 | < .001 |
| g. Period, random slope, and IU model (Model 4) | 5818 | -7563.283 | 15142.570 | 15195.920 |  |  |  |  |
| h. Period, random slope, IU, and interaction model (Model 5) | 5818 | -7562.946 | 15143.890 | 15203.920 |  |  |  |  |
|  |  |  |  |  |  |  |  |  |
| Parental warmth |  |  |  |  |  |  |  |  |
| a. Unconditional model | 1598 | -1723.105 | 3452.210 | 3468.340 |  |  |  |  |
| b. Unconditional model AR individual (Model 1) | 1598 | -1709.779 | 3427.558 | 3449.064 | a vs b | 26.652 | 1 | < .001 |
| c. Unconditional model CAR individual | 1598 | -1709.779 | 3427.558 | 3449.064 |  |  |  |  |
| d. Unconditional model AR individual and family (Model 1b) | 1598 | -1709.399 | 3428.797 | 3455.680 | b vs d | 0.761 | 1 | .383 |
| e. Period model (Model 2) | 1598 | -1709.747 | 3429.493 | 3456.376 |  |  |  |  |
| f. Period and random slope model (Model 3) | 1598 | -1698.497 | 3410.994 | 3448.629 | e vs f | 22.499 | 2 | < .001 |
| g. Period, random slope, gender, and IU model (Model 4) | 1532 | -1636.048 | 3290.095 | 3338.104 |  |  |  |  |
| h. Period, random slope, gender, IU, and interaction model (Model 5) | 1532 | -1635.928 | 3291.857 | 3345.200 |  |  |  |  |
|  |  |  |  |  |  |  |  |  |
| Parental criticism |  |  |  |  |  |  |  |  |
| a. Unconditional model | 1598 | -2534.510 | 5075.021 | 5091.150 |  |  |  |  |
| b. Unconditional model AR PPN (Model 1) | 1598 | -2518.527 | 5045.054 | 5066.560 | a vs b | 31.967 | 1 | < .001 |
| c. Unconditional model CAR individual | 1598 | -2518.527 | 5045.054 | 5066.560 |  |  |  |  |
| d. Unconditional model AR individual and family (Model 1b) | 1598 | -2515.812 | 5041.624 | 5068.506 | b vs d | 5.430 | 1 | .020 |
| e. Period model (Model 2) | 1598 | -2513.892 | 5039.784 | 5072.043 |  |  |  |  |
| f. Period and random slope model (Model 3) | 1598 | -2494.128 | 5008.257 | 5062.022 | e vs f | 39.527 | 4 | < .001 |
| g. Period, random slope, gender, and IU model (Model 4) | 1532 | -2388.684 | 4801.369 | 4865.381 |  |  |  |  |
| h. Period, random slope, gender, IU, and interaction model (Model 5) | 1532 | -2388.247 | 4802.495 | 4871.841 |  |  |  |  |
