# Supplementary material for: Does the COVID-19 pandemic impact parents’ and adolescents’ well-being? An EMA-study on daily affect and parenting
Source: PLoS One. 2020 Oct 16;15(10):e0240962. doi: 10.1371/journal.pone.0240962 (PMC7567366; doi:10.1371/journal.pone.0240962)
Supplement: S5 Table — Table A: Model results on the relation between period and negative affect, and the moderating role of intolerance of uncertainty in adolescents. Table B: Model results on the relation between period and positive affect, and the moderating role of intolerance of uncertainty in adolescents. Table C: Model results on the relation between period and parental criticism, and the moderating role of intolerance of uncertainty in adolescents. Table D: Model results on the relation between period and parental warmth, and the moderating role of intolerance of uncertainty in adolescents. (DOCX) [file pone.0240962.s009.docx]

**S5 Table. Model results adolescents.**

**Table A. Results of Model 1, Model 2, Model3, and Model 4 on the Relation Between Period and Negative Affect and the Moderating Role of Intolerance of Uncertainty in Adolescents*.***

|  | Model 1 | | | |  | Model 2 | | | | |  | | Model 3 | | | |  | | Model 4 | | | | |
| --- | --- | --- | --- | --- | --- | --- | --- | --- | --- | --- | --- | --- | --- | --- | --- | --- | --- | --- | --- | --- | --- | --- | --- |
|  | *B* | *SE* | *t* | *p* |  | *B* | *SE* | *t* | *p* |  | | *B* | | *SE* | *t* | *p* | |  | | *B* | *SE* | *t* | *p* |
| Intercept | 1.415 | .076 | 18.622 | < .001 |  | 1.408 | .077 | 18.267 | < .001 |  | | 1.406 | | .083 | 17.014 | < .001 | |  | | 1.418 | .078 | 18.164 | < .001 |
| Period (baseline vs COVID-19) |  |  |  |  |  | 0.016 | .027 | 0.595 | .552 |  | | 0.025 | | .049 | .509 | .611 | |  | | 0.033 | .052 | 0.638 | .524 |
| IU |  |  |  |  |  |  |  |  |  |  | |  | |  |  |  | |  | | 0.030 | .011 | 2.737 | .010 |
| Random effects |  |  |  |  |  |  |  |  |  |  | |  | |  |  |  | |  | |  |  |  |  |
| Between-person variance | 0.189 |  |  |  |  | 0.190 |  |  |  |  | | 0.221 | |  |  |  | |  | | 0.183 |  |  |  |
| Within-person variance | 0.400 |  |  |  |  | 0.400 |  |  |  |  | | 0.386 | |  |  |  | |  | | 0.391 |  |  |  |
| Random effect variance |  |  |  |  |  |  |  |  |  |  | | 0.058 | |  |  |  | |  | | 0.062 |  |  |  |
| ICC individual | 0.321 |  |  |  |  |  |  |  |  |  | |  | |  |  |  | |  | |  |  |  |  |
|  |  |  |  |  |  |  |  |  |  |  | |  | |  |  |  | |  | |  |  |  |  |
| N adolescents | 34 |  |  |  |  | 34 |  |  |  |  | | 34 | |  |  |  | |  | | 32 |  |  |  |
| N observations | 2653 |  |  |  |  | 2653 |  |  |  |  | | 2653 | |  |  |  | |  | | 2497 |  |  |  |

**Table B. Results of Model 1, Model 2, Model3, and Model 4 on the Relation Between Period and Positive Affect and the Moderating Role of Intolerance of Uncertainty in Adolescents.**

|  | Model 1 | | | |  | Model 2 | | | |  | | Model 3 | | | |  | | Model 4 | | | |
| --- | --- | --- | --- | --- | --- | --- | --- | --- | --- | --- | --- | --- | --- | --- | --- | --- | --- | --- | --- | --- | --- |
|  | *B* | *SE* | *t* | *p* |  | *B* | *SE* | *t* | *p* | |  | *B* | *SE* | *t* | *p* | |  | *B* | *SE* | *t* | *p* |
| Intercept | 5.559 | .107 | 51.736 | < .001 |  | 5.548 | .109 | 50.812 | < .001 | |  | 5.557 | .114 | 48.701 | < .001 | |  | 5.516 | .106 | 52.240 | < .001 |
| Period (baseline vs COVID-19) |  |  |  |  |  | 0.025 | .043 | 0.574 | .566 | |  | -0.010 | .104 | -0.097 | .923 | |  | -0.008 | .111 | -0.073 | .942 |
| IU |  |  |  |  |  |  |  |  |  | |  |  |  |  |  | |  | -0.044 | .015 | -2.917 | .007 |
| Random effects |  |  |  |  |  |  |  |  |  | |  |  |  |  |  | |  |  |  |  |  |
| Between-person variance | 0.375 |  |  |  |  | 0.375 |  |  |  | |  | 0.418 |  |  |  | |  | 0.332 |  |  |  |
| Within-person variance | 0.756 |  |  |  |  | 0.755 |  |  |  | |  | 0.682 |  |  |  | |  | 0.675 |  |  |  |
| Random effect variance |  |  |  |  |  |  |  |  |  | |  | 0.314 |  |  |  | |  | 0.339 |  |  |  |
| ICC individual | 0.332 |  |  |  |  |  |  |  |  | |  |  |  |  |  | |  |  |  |  |  |
|  |  |  |  |  |  |  |  |  |  | |  |  |  |  |  | |  |  |  |  |  |
| N adolescents | 34 |  |  |  |  | 34 |  |  |  | |  | 34 |  |  |  | |  | 32 |  |  |  |
| N observations | 2653 |  |  |  |  | 2653 |  |  |  | |  | 2653 |  |  |  | |  | 2497 |  |  |  |

**Table C. Results of Model 1, Model 2, Model3, and Model 4 on the Relation Between Period and Parental Criticism and the Moderating Role of Intolerance of Uncertainty in Adolescents.**

|  | Model 1 | | | |  | Model 1b | | | |  | Model 2 | | | |  | Model 3 | | | |  | Model 4 | | | | |
| --- | --- | --- | --- | --- | --- | --- | --- | --- | --- | --- | --- | --- | --- | --- | --- | --- | --- | --- | --- | --- | --- | --- | --- | --- | --- |
|  | *B* | *SE* | *t* | *p* |  | *B* | *SE* | *t* | *p* |  | *B* | *SE* | *t* | *p* |  | *B* | *SE* | *t* | *p* |  | *B* | *SE* | *t* | *p* |  |
| Intercept | 1.985 | .148 | 13.412 | < .001 |  | 1.985 | .148 | 13.397 | < .001 |  | 1.969 | .151 | 13.036 | < .001 |  | 1.961 | .152 | 12.943 | < .001 |  | 2.043 | .157 | 12.976 | < .001 |  |
| Period (baseline vs COVID-19) |  |  |  |  |  |  |  |  |  |  | 0.036 | .062 | 0.576 | .565 |  | 0.110 | .135 | .814 | .416 |  | 0.120 | .137 | 0.877 | .381 |  |
| Gender parents |  |  |  |  |  |  |  |  |  |  |  |  |  |  |  |  |  |  |  |  | -0.121 | .058 | -2.099 | .036 |  |
| IU |  |  |  |  |  |  |  |  |  |  |  |  |  |  |  |  |  |  |  |  | 0.028 | .024 | 1.208 | .237 |  |
| Random effects |  |  |  |  |  |  |  |  |  |  |  |  |  |  |  |  |  |  |  |  |  |  |  |  |  |
| Between-person variance | 0.704 |  |  |  |  | 0.706 |  |  |  |  | 0.707 |  |  |  |  | 0.725 |  |  |  |  | 0.714 |  |  |  |  |
| Within-person variance | 0.859 |  |  |  |  | 0.859 |  |  |  |  | 0.859 |  |  |  |  | 0.759 |  |  |  |  | 0.765 |  |  |  |  |
| Random effect variance |  |  |  |  |  |  |  |  |  |  |  |  |  |  |  | 0.498 |  |  |  |  | 0.475 |  |  |  |  |
| ICC individual | .450 |  |  |  |  | 0.451 |  |  |  |  |  |  |  |  |  |  |  |  |  |  |  |  |  |  |  |
|  |  |  |  |  |  |  |  |  |  |  |  |  |  |  |  |  |  |  |  |  |  |  |  |  |  |
| Parent variance |  |  |  |  |  | 0.000 |  |  |  |  |  |  |  |  |  |  |  |  |  |  |  |  |  |  |  |
| Random effect variance |  |  |  |  |  |  |  |  |  |  |  |  |  |  |  |  |  |  |  |  |  |  |  |  |  |
| ICC parent |  |  |  |  |  | 0.000 |  |  |  |  |  |  |  |  |  |  |  |  |  |  |  |  |  |  |  |
|  |  |  |  |  |  |  |  |  |  |  |  |  |  |  |  |  |  |  |  |  |  |  |  |  |  |
| N adolescents | 34 |  |  |  |  | 34 |  |  |  |  | 34 |  |  |  |  | 34 |  |  |  |  | 32 |  |  |  |  |
| N parents |  |  |  |  |  | 67 |  |  |  |  |  |  |  |  |  |  |  |  |  |  |  |  |  |  |  |
| N observations | 1385 |  |  |  |  | 1385 |  |  |  |  | 1385 |  |  |  |  | 1385 |  |  |  |  | 1302 |  |  |  |  |

**Table D. Results of Model 1, Model 2, Model3, and Model 4 on the Relation Between Period and Parental Warmth and the Moderating Role of Intolerance of Uncertainty in Adolescents.**

|  | Model 1 | | | |  | Model 1b | | | |  | Model 2 | | | |  | Model 3 | | | |  | | Model 4 | | | | |
| --- | --- | --- | --- | --- | --- | --- | --- | --- | --- | --- | --- | --- | --- | --- | --- | --- | --- | --- | --- | --- | --- | --- | --- | --- | --- | --- |
|  | *B* | *SE* | *t* | *p* |  | *B* | *SE* | *t* | *p* |  | *B* | *SE* | *t* | *p* |  | *B* | *SE* | *t* | *p* |  | *B* | | *SE* | *t* | *p* |  |
| Intercept | 5.770 | .165 | 35.044 | < .001 |  | 5.768 | .166 | 34.761 | < .001 |  | 5.756 | .167 | 34.365 | < .001 |  | 5.765 | .164 | 35.199 | < .001 |  | 5.710 | | .170 | 33.568 | < .001 |  |
| Period (baseline vs COVID-19) |  |  |  |  |  |  |  |  |  |  | 0.026 | .051 | 0.500 | .617 |  | -0.016 | .108 | -0.148 | .882 |  | -0.036 | | .113 | -0.319 | .750 |  |
| Gender parents |  |  |  |  |  |  |  |  |  |  |  |  |  |  |  |  |  |  |  |  | 0.014 | | .077 | 0.184 | .855 |  |
| IU |  |  |  |  |  |  |  |  |  |  |  |  |  |  |  |  |  |  |  |  | -0.033 | | .025 | -1.314 | .199 |  |
| Random effects |  |  |  |  |  |  |  |  |  |  |  |  |  |  |  |  |  |  |  |  |  | |  |  |  |  |
| Between-person variance | 0.892 |  |  |  |  | 0.883 |  |  |  |  | 0.882 |  |  |  |  | 0.822 |  |  |  |  | 0.787 | |  |  |  |  |
| Within-person variance | 0.594 |  |  |  |  | 0.569 |  |  |  |  | 0.569 |  |  |  |  | 0.487 |  |  |  |  | 0.503 | |  |  |  |  |
| Random effect variance |  |  |  |  |  |  |  |  |  |  |  |  |  |  |  | 0.302 |  |  |  |  | 0.314 | |  |  |  |  |
| ICC individual | 0.600 |  |  |  |  | 0.588 |  |  |  |  |  |  |  |  |  |  |  |  |  |  |  | |  |  |  |  |
|  |  |  |  |  |  |  |  |  |  |  |  |  |  |  |  |  |  |  |  |  |  | |  |  |  |  |
| Parent variance |  |  |  |  |  | 0.049 |  |  |  |  | 0.049 |  |  |  |  | 0.104 |  |  |  |  | 0.110 | |  |  |  |  |
| Random effect variance |  |  |  |  |  |  |  |  |  |  |  |  |  |  |  | 0.027 |  |  |  |  | 0.027 | |  |  |  |  |
| ICC parent |  |  |  |  |  | 0.033 |  |  |  |  |  |  |  |  |  |  |  |  |  |  |  | |  |  |  |  |
|  |  |  |  |  |  |  |  |  |  |  |  |  |  |  |  |  |  |  |  |  |  | |  |  |  |  |
| N adolescents | 34 |  |  |  |  | 34 |  |  |  |  | 34 |  |  |  |  | 34 |  |  |  |  | 32 | |  |  |  |  |
| N parents |  |  |  |  |  | 67 |  |  |  |  | 67 |  |  |  |  | 67 |  |  |  |  | 63 | |  |  |  |  |
| N observations | 1385 |  |  |  |  | 1385 |  |  |  |  | 1385 |  |  |  |  | 1385 |  |  |  |  | 1302 | |  |  |  |  |
