# Supplementary material for: Does the COVID-19 pandemic impact parents’ and adolescents’ well-being? An EMA-study on daily affect and parenting
Source: PLoS One. 2020 Oct 16;15(10):e0240962. doi: 10.1371/journal.pone.0240962 (PMC7567366; doi:10.1371/journal.pone.0240962)
Supplement: S6 Table — (DOCX) [file pone.0240962.s010.docx]

**S6 Table. Model fit statistics of all models of adolescents.**

|  |  |  |  |  | ΔLL test | | | |
| --- | --- | --- | --- | --- | --- | --- | --- | --- |
| Title | Observations | LL | AIC | BIC | Comparison | Δ-2LL | *df* | *p* |
| Negative affect |  |  |  |  |  |  |  |  |
| a. Unconditional model | 2653 | -2608.002 | 5222.005 | 5239.665 |  |  |  |  |
| b. Unconditional model AR individual (Model 1) | 2653 | -2595.711 | 5199.422 | 5222.956 | a vs b | 24.583 | 1 | < .001 |
| c. Unconditional model CAR individual | 2653 | -2595.711 | 5199.422 | 5222.956 |  |  |  |  |
| d. Period model (Model 2) | 2653 | -2595.534 | 5201.067 | 5230.485 |  |  |  |  |
| e. Period and random slope model (Model 3) | 2653 | -2575.654 | 5165.308 | 5206.492 | d vs e | 39.759 | 2 | < .001 |
| f. Period, random slope, and IU model (Model 4) | 2497 | -2437.503 | 4891.007 | 4937.589 |  |  |  |  |
| g. Period, random slope, IU, and interaction model (Model 5) | 2497 | -2437.186 | 4892.372 | 4944.778 |  |  |  |  |
|  |  |  |  |  |  |  |  |  |
| Positive affect |  |  |  |  |  |  |  |  |
| a. Unconditional model | 2653 | -3443.924 | 6893.849 | 6911.499 |  |  |  |  |
| b. Unconditional model AR individual (Model 1) | 2653 | -3365.416 | 6738.832 | 6762.365 | a vs b | 157.017 | 1 | < .001 |
| c. Unconditional model CAR individual | 2653 | -3365.416 | 6738.832 | 6762.365 |  |  |  |  |
| d. Period model (Model 2) | 2653 | -3365.252 | 6740.503 | 6769.920 |  |  |  |  |
| e. Period and random slope model (Model 3) | 2653 | -3313.353 | 6640.705 | 6681.889 | d vs e | 103.798 | 2 | < .001 |
| f. Period, random slope, and IU model (Model 4) | 2497 | -3102.189 | 6220.337 | 6266.960 |  |  |  |  |
| g. Period, random slope, IU, and interaction model (Model 5) | 2497 | -3102.169 | 6222.338 | 6274.743 |  |  |  |  |
|  |  |  |  |  |  |  |  |  |
| Parental warmth |  |  |  |  |  |  |  |  |
| a. Unconditional model | 1385 | -1662.053 | 3330.106 | 3345.807 |  |  |  |  |
| b. Unconditional model AR individual (Model 1) | 1385 | -1615.318 | 3238.636 | 3259.570 | a vs b | 93.470 | 1 | < .001 |
| c. Unconditional model CAR individual | 1385 | -1615.318 | 3238.635 | 3259.569 |  |  |  |  |
| d. Unconditional model AR individual and parent (Model 1b) | 1385 | -1602.661 | 3215.322 | 3241.489 | b vs d | 25.314 | 1 | < .001 |
| e. Period model (Model 2) | 1385 | -1602.537 | 3217.074 | 3248.475 |  |  |  |  |
| f. Period and random slope model (Model 3) | 1385 | -1565.122 | 3150.244 | 3202.578 | e vs f | 74.831 | 4 | < .001 |
| g. Period, random slope, gender parent, and IU model (Model 4) | 1302 | -1490.693 | 3005.385 | 3067.445 |  |  |  |  |
| h. Period, random slope, gender parent, IU, and interaction (Model 5) | 1302 | -1490.503 | 3007.007 | 3074.238 |  |  |  |  |
|  |  |  |  |  |  |  |  |  |
| Parental criticism |  |  |  |  |  |  |  |  |
| a. Unconditional model | 1385 | -1906.684 | 3819.368 | 3835.069 |  |  |  |  |
| b. Unconditional model AR individual (Model 1) | 1385 | -1862.954 | 3733.908 | 3754.842 | a vs b | 87.460 | 1 | < .001 |
| c. Unconditional model CAR individual | 1385 | -1862.954 | 3733.908 | 3754.842 |  |  |  |  |
| d. Unconditional model AR individual and parent (Model 1b) | 1385 | -1861.491 | 3732.983 | 3759.150 | b vs d | 2.925 | 1 | .087 |
| e. Period model (Model 2) | 1385 | -1862.788 | 3735.577 | 3761.744 |  |  |  |  |
| f. Period and random slope model (Model 3) | 1385 | -1835.823 | 3685.645 | 3722.279 | e vs f | 53.931 | 2 | < .001 |
| g. Period, random slope, gender parent, and IU model (Model 4) | 1302 | -1731.690 | 3481.380 | 3527.927 |  |  |  |  |
| h. Period, random slope, gender parent, IU, and interaction (Model 5) | 1302 | -1731.686 | 3483.373 | 3535.089 |  |  |  |  |
