# Supplementary material for: Does the COVID-19 pandemic impact parents’ and adolescents’ well-being? An EMA-study on daily affect and parenting
Source: PLoS One. 2020 Oct 16;15(10):e0240962. doi: 10.1371/journal.pone.0240962 (PMC7567366; doi:10.1371/journal.pone.0240962)
Supplement: S7 Table — (DOCX) [file pone.0240962.s011.docx]

**S7 Table. Overview of EMA items used in the current study.**

*Adolescents*

| **Item** | **Answer category** |
| --- | --- |
| How do you feel at the moment? - Happy | Likert type (1 = not at all happy to 7 = very happy) |
| How do you feel at the moment? - Sad | Likert type (1 = not at all sad to 7 = very sad) |
| How do you feel at the moment? - Relaxed | Likert type (1 = not at all relaxed to 7 = very relaxed) |
| How do you feel at the moment? - Irritated | Likert type (1 = not at all irritated to 7 = very irritated) |
| To whom did you talk today? | Multiple options were possible: 1 = mother, 2 = father, 3 = stepmother, 4 = stepfather, 5 = nobody |
| Throughout the day, how critical was your (mother/father/stepmother/stepfather) towards you? | Likert type (1 = not at all to 7 = very) |
| Throughout the day, how warm/loving was your (mother/father/stepmother/stepfather) towards you? | Likert type (1 = not at all to 7 = very) |
| *What was helpful today at home? | Multiple options were possible:1 = homework, 2 = watching television/series, 3 = listening to music, 4 = gaming, 5 = watch social media, 6 = reading a book, 7 = sports, 8 = chilling, 9 = online contact with relatives or friends, 10 = being together with the family, 11 = playing card/board games, 12 = DIY or crafts, 13 = cooking/dining, 14 = something else, namely [TEXT] |
| *What made it difficult today at home? | 1 = boredom, 2= conflicts, 3= homework, 4 = irritations with family members, 5 = noise disturbance, 6 = loneliness, 7 = missing social contact with friends, 8 = worrying about own health, 9 = worrying about the health of others, 10 = concerns about the coronavirus in general, 11 = coronavirus-related news items, 12 = something else, namely [TEXT] |

* only presented in EMA during the COVID-19 pandemic.

*Parents*

| **Item** | **Answer category** |
| --- | --- |
| How do you feel at the moment? - Happy | Likert type (1 = not at all happy to 7 = very happy) |
| How do you feel at the moment? - Sad | Likert type (1 = not at all sad to 7 = very sad) |
| How do you feel at the moment? - Relaxed | Likert type (1 = not at all relaxed to 7 = very relaxed) |
| How do you feel at the moment? - Irritated | Likert type (1 = not at all irritated to 7 = very irritated) |
| Did you talk to your child today? | 1 = yes, 2 = no |
| Throughout the day, how critical were you towards your child? | Likert type (1 = not at all to 7 = very) |
| Throughout the day, how warm/loving were you towards your child? | Likert type (1 = not at all to 7 = very) |
| *What was helpful today at home? | Multiple options were possible:1 = work, 2 = watching television/series, 3 = listening to music, 4 = gaming, 5 = watch social media, 6 = reading a book, 7 = sports, 8 = chilling, 9 = online contact with relatives or friends, 10 = being together with the family, 11 = playing card/board games, 12 = DIY or crafts, 13 = cooking/dining, 14 = meditation/mindfulness, 15 = something else, namely [TEXT] |
| *What made it difficult today at home? | 1 = boredom, 2= conflicts, 3= work, 4 = irritations with family members, 5 = noise disturbance, 6 = loneliness, 7 = missing social contact with friends, 8 = worrying about own health, 9 = worrying about the health of others, 10 = concerns about the coronavirus in general, 11 = coronavirus-related news items, 12 = something else, namely [TEXT] |

* only presented in EMA during the COVID-19 pandemic.
