# Supplementary material for: Does the COVID-19 pandemic impact parents’ and adolescents’ well-being? An EMA-study on daily affect and parenting
Source: PLoS One. 2020 Oct 16;15(10):e0240962. doi: 10.1371/journal.pone.0240962 (PMC7567366; doi:10.1371/journal.pone.0240962)
Supplement: S8 Table — (DOCX) [file pone.0240962.s012.docx]

**S8 Table. Overview of EMA items of the exit questionnaire used in the current study.**

*Adolescents*

| **Item** | **Answer category** |
| --- | --- |
| Do you know or do you think you have (had) COVID-19? | 1 = No, I (probably) have not had it, 2 = Yes, I (probably) have had it before the EMA during the COVID-19 pandemic, 3 = Yes, I (probably) have had it during the EMA during the COVID-19 pandemic |

*Parents*

| **Item** | **Answer category** |
| --- | --- |
| Do you know or do you think you have (had) COVID-19? | 1 = No, I (probably) have not had it, 2 = Yes, I (probably) have had it before the EMA during the COVID-19 pandemic, 3 = Yes, I (probably) have had it during the EMA during the COVID-19 pandemic |
| What was the most stressful in the past two weeks (EMA period)? | 1 = boredom, 2= conflicts, 3= work, 4 = irritation with family members, 5 = noise disturbance, 6 = loneliness, 7 = missing social contact with friends, 8 = worrying about own health, 9 = worrying about the health of others, 10 = illness of a loved one, 11 = concerns about the coronavirus in general, 12 = coronavirus-related news items, 13 = finances, 14 = something else, namely [TEXT] |
| During the past two weeks (EMA period), how much time did you spend on average per day with homeschooling one or more children? | 1 = 0, 2 = 0-2, 3 = 3-5, 4 = 6-8 |
| During the past two weeks (EMA period), how many hours per week did you work outside your home? | 1 = 0, 2 = 0-16, 3 = 17-32, 4 = 33-40, 5 = >40 |
| Does the number of hours deviate from the situation before the corona crisis? Complete the sentence: In the past two weeks I worked ... outside home than usual | 1 = More, 2 = Less, 3 = Equally |
| During the past two weeks (EMA period), did you have direct contact with corona infected persons (patient contact) at work? |  |
| During the past two weeks (EMA period), how many hours per week did you work from home? | 1 = 0, 2 = 0-16, 3 = 17-32, 4 = 33-40, 5 = >40 |
| Does the number of hours deviate from the situation before the corona crisis? Complete the sentence: In the past two weeks I worked ... at home than usual | 1 = More, 2 = Less, 3 = Equally |
